# An ISA-TAB-Nano based data collection framework to support data-driven modelling of nanotoxicology

**DOI:** 10.3762/bjnano.6.202

**Published:** 2015-10-05

**Authors:** Richard L Marchese Robinson, Mark T D Cronin, Andrea-Nicole Richarz, Robert Rallo

**Affiliations:** 1 School of Pharmacy and Biomolecular Sciences, Liverpool John Moores University, James Parsons Building, Byrom Street, Liverpool, L3 3AF, United Kingdom; 2 Departament d'Enginyeria Informatica i Matematiques, Universitat Rovira i Virgili, Av. Paisos Catalans 26, 43007 Tarragona, Catalunya, Spain

**Keywords:** databases, ISA-TAB-Nano, nanoinformatics, nanotoxicology, quantitative structure–activity relationship (QSAR)

## Abstract

Analysis of trends in nanotoxicology data and the development of data driven models for nanotoxicity is facilitated by the reporting of data using a standardised electronic format. ISA-TAB-Nano has been proposed as such a format. However, in order to build useful datasets according to this format, a variety of issues has to be addressed. These issues include questions regarding exactly which (meta)data to report and how to report them. The current article discusses some of the challenges associated with the use of ISA-TAB-Nano and presents a set of resources designed to facilitate the manual creation of ISA-TAB-Nano datasets from the nanotoxicology literature. These resources were developed within the context of the NanoPUZZLES EU project and include data collection templates, corresponding business rules that extend the generic ISA-TAB-Nano specification as well as Python code to facilitate parsing and integration of these datasets within other nanoinformatics resources. The use of these resources is illustrated by a “Toy Dataset” presented in the Supporting Information. The strengths and weaknesses of the resources are discussed along with possible future developments.

## Introduction

Nanotechnology, which may be considered the design and application of engineered nanomaterials with desired properties [1–2], is of increasing importance [3–4]. Nanomaterials may be considered to be any chemicals with (a majority of) constituent particles with one or more dimensions in the nanoscale (typically 1–100 nm) range and engineered nanomaterials may be considered to be any nanomaterials that are intentionally produced. (It should be noted that slightly different definitions of these terms have been proposed by different organisations [1] and the European Commission has recommended a specific definition of a “nanomaterial” for legislative and policy purposes within the European Union [5].)

Nanomaterials have been used and/or have been investigated for use in a diverse range of applications such as sunscreens, cosmetics, electronics and medical applications [2,4,6–7]. In addition to interest in the benefits offered by nanotechnology, concerns have also been raised about the potential risk posed by nanomaterials to human health and the environment [3–4,7]. Various research initiatives have been (and are being) funded to advance scientific understanding of nanotechnology and nanosafety and to enable the appropriate selection, design and regulation of nanomaterials for technological applications [3,8–9]. There is a particular interest in the possibility of using computational approaches as part of the safety assessment of nanomaterials, e.g., to enable “safety by design” [3,7,9–10].

Experimental data are critical to advancing understanding of the properties of nanomaterials and the ability to design nanomaterials with desirable technological properties and acceptable safety profiles [2,9–11]. In order to enable “safety by design”, data from toxicity studies need to be related to relevant structural/physicochemical data [10], where the latter may include information about chemical composition as well as a range of other measured properties such as size distribution statistics and zeta potential, to name but two [12]. Being able to relate these data allows for the development of predictive models based on quantitative structure–activity relationships (QSARs) for nanomaterials – so-called quantitative nanostructure–activity relationships (“QNARs”) [10] or “nano-QSARs” [13] – as well as “category formation” and “read-across” predictions [9,14–15].

In order to make most effective use of these data, experimental datasets should be made available via a standardised, electronic format that facilitates meaningful exchange of information between different researchers, submission to (web-based) searchable databases, integration with other electronic data resources and analysis via appropriate (modelling) software [9,16–18]. This could entail directly populating files based on a standardised format or direct entry of data into searchable databases using a (web-based) data entry tool [19], followed via data export/exchange in a standardised format. However, in contrast to directly populating standardised, structured files (such as spreadsheets), direct entry of data into (web-based) searchable databases may not be possible for domain experts (e.g., nanotoxicologists in experimental labs) with little or no informatics support. These researchers may not have their own, in-house database systems and data entry to a third party database at the point of data collection may not be practical. Data collected using standardised, structured files may be readily, programatically submitted to (web-based) searchable databases at a later stage in the research cycle.

Standardised, structured files also facilitate programmatic analysis (i.e., entirely new codes and/or configuration files do not need to be developed for each new dataset) for the purposes of computational modelling. They also facilitate integration between datasets, partly due to the ease of programmatic analysis and in part because standardisation makes it clearer when two items of (meta)data in distinct datasets are related. Data integration within searchable databases supports computational modelling via enabling data from multiple sources to be combined, in principle, for more robust, generalisable analysis and via facilitating the identification of data which are relevant to the needs of a given modeller.

Regarding the nature of these standardised, structured files, whilst more complicated file formats based on the eXtended Markup Language (XML) or the Resource Description Framework (RDF) might be considered, a spreadsheet-based file format offers a key advantage: most scientists are likely to be familiar with creating, editing and viewing spreadsheet-based datasets [17,20–21]. Indeed, these kinds of files can be edited and viewed using widely used, non-specialist software (such as Microsoft Excel), whilst (to some extent) a spreadsheet-like interface may be retained within specialist software designed to ensure the files are compliant with the rules of a standardised specification [17,20,22]. However, no claim is being made as to the intrinsic optimality of a spreadsheet-based format: a detailed discussion of the advantages and disadvantages of different file formats is beyond the scope of the current publication and interested readers are referred to the cited literature and the references therein [17,20–21].

The ISA-TAB-Nano specification, comprising a set of interrelated spreadsheet-based tabular file types, was recently proposed as a solution to the requirement for a standardised, electronic format for nanomaterial data [16–17,23]. However, as well as a general specification specifying how different kinds of (meta)data should be recorded in a standardised fashion, additional requirements for nanotoxicology datasets to be most valuable for analysis of trends and development of data driven models exist. These requirements include the need to report the necessary physicochemical parameters, experimental details and other relevant metadata such as provenance [12,24–27]. Whilst the generic ISA-TAB-Nano specification [17,23] specifically calls for relevant provenance information to be provided, and facilitates presentation of other (meta)data, it does not specify all of the (meta)data which should be recorded nor exactly how these (meta)data should be presented.

This article presents a set of resources which were designed for manually harvesting data from the published literature to create ISA-TAB-Nano datasets in order to support analysis and modelling of nanotoxicology data, including the integration of these data within online, searchable databases. Specifically, these resources are as follows: a collection of Excel templates for creating ISA-TAB-Nano files containing specific, relevant (meta)data manually harvested from the scientific literature; a corresponding set of business rules for populating these templates which build upon the generic ISA-TAB-Nano specification; a Python program for converting the resulting ISA-TAB-Nano files to tab-delimited text files to facilitate computational analysis and database submission. Since there is a growing interest in the use of ISA-TAB-Nano as a community standard for organising nanomaterial data, from a variety of individual researchers and organizations [3,28–32], it is anticipated that these resources will be of value for the research community.

These resources were developed within the context of the NanoPUZZLES project [33], but their development was informed via discussions with various researchers in the nanoinformatics/nanotoxicology community and consideration of various complementary nanoinformatics resources such as those developed within the MODERN [34] and eNanoMapper [35] projects.

The rest of the article is organised as follows. Section 1 of “Results and Discussion” provides a brief overview of the generic ISA-TAB-Nano specification. Section 2 summarises some challenges associated with the use of this generic specification (especially when used to collect data from the literature), which the current work sought to address. Section 3 summarises the data collection templates and the basis on which they were developed. Section 4 summarises the new business rules which were created for populating these templates. Section 5 provides an overview of the Python program written to facilitate analysis and databases submission of datasets created using these templates. Section 6 presents a “Toy Dataset” created using these templates. Section 7 presents a critical appraisal of the developed resources, discusses links to related research initiatives and resources along with possible future directions for this work. The “take home” messages of this article are summarised under “Conclusion”. The challenges, business rules and notable limitations of the presented resources (summarised in sections 2, 4 and 7, respectively) are fully explained in the Supporting Information. The resources described in this article, along with the “Toy Dataset”, are publicly available under open licenses (see Supporting Information Files 1–4).

## Results and Discussion

### 1 A brief overview of the generic ISA-TAB-Nano specification

The ISA-TAB-Nano specification [17,23] extends the ISA-TAB specification [18,20,22,36] which was previously proposed as an exchange standard for biological data and metadata based on a standardised metadata representation. Unless noted otherwise, the specification incorporates [17,23] all the business rules (e.g., restrictions on which fields can hold multiple values) associated with the original ISA-TAB specification [36]. The official ISA-TAB-Nano wiki [23] provides the most up to date information regarding the generic ISA-TAB-Nano specification, including detailed descriptions [37–40] and Excel templates for each of the file types described below. Since the original description of the specification in Thomas et al. [17], two revisions (version 1.1 and version 1.2) of the specification had been published on the wiki at the time of writing. The overview provided in the current paper refers to version 1.2 of ISA-TAB-Nano. Since the specification is extensively described elsewhere [17,23], the following overview focuses on the essential background required to understand the following sections of the current paper.

**Figure 1:**
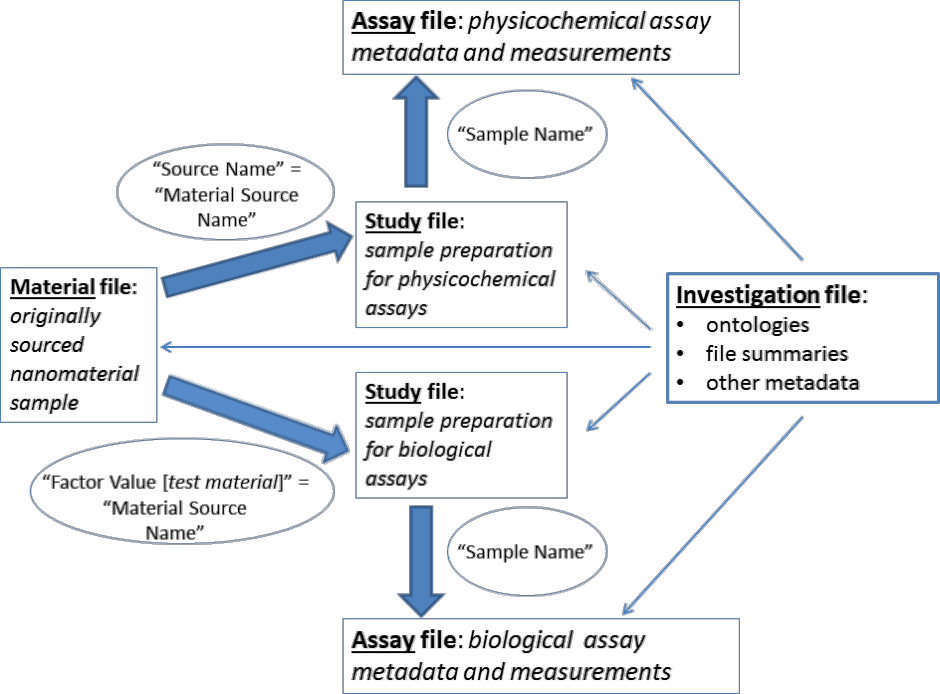
A schematic illustration of the links between ISA-TAB-Nano files. Biological or material samples are prepared for measurements in biological or physicochemical assays respectively. Assay files link measurement values with prepared sample identifiers (“Sample Name” values). Study files describe sample preparation. Material files describe the nanomaterials obtained for testing, denoted via their “Material Source Name” identifiers. N.B. Italic font denotes generic names, e.g., “Factor Value [*test material*]” is replaced with “Factor Value [nanomaterial]” in the NanoPUZZLES in vitro cell-based Study file template.

The ISA-TAB-Nano specification describes a set of four linked file types (Investigation, Study, Assay, Material), each of which is a spreadsheet-like table, which are used to record different kinds of (meta)data associated with a given “investigation”, which may be considered to correspond to a set of different kinds of experimental studies carried out on a given set of nanomaterials [36]. In addition, the specification describes corresponding business rules governing how these files can be populated. A given “investigation” is associated with a single Investigation file and, potentially, multiple Study, Assay and Material files. The kinds of (meta)data each file type is designed to record and the links between different kinds of files is summarised in Figure 1 and discussed in more detail below.

#### Investigation file

The Investigation file [37] reports key metadata describing the terms used in the other files as well as reporting overall conclusions derived from the “investigation”, if any.

#### Material file

Each of the nanomaterial samples (implicitly as originally sourced for the “investigation” [17]) is described by a corresponding Material file [40] associated with a unique identifier reported in the “Material Source Name” column and used to label the Material file. A Material file presents chemical composition information along with other descriptive information about the sample such as nominal or manufacturer supplied characteristics reported via end user defined “Characteristics [*characteristic name*]” columns. Since nanomaterials of diverse types (e.g., dendrimers, carbon nanotubes, surface-coated metal oxides) may comprise different components (e.g., core and shell), the initial rows of the Material file are used to describe the overall nanomaterial sample with subsequent rows used to describe the individual components: the overall sample and different components are each assigned unique values in the “Material Name” column.

#### Study file

A Study file [38] describes the preparation of samples for analysis via some assay protocol. The identifiers of prepared samples are reported in “Sample Name” columns, with sequentially prepared samples corresponding to identifiers in sequential “Sample Name” columns, and the identifier(s) of the original material(s) from which these samples were prepared is (are) reported in the “Source Name” column. In principle, multiple “Source Name” identifiers might correspond to one or more “Sample Name” identifiers [36]. However, in the simplest case (as adopted in the current work), a single prepared sample corresponds to a single original material, i.e., each row corresponds to a single “Source Name” and a single “Sample Name” identifier. Properties associated with the original material or, more specifically, a prepared sample may be reported via “Characteristics [*characteristic name*]” columns situated after the “Source Name” column or after the relevant “Sample Name” column respectively. Here, it should be noted that the properties recorded via these columns should not include experimental endpoints which would be reported via an Assay file or other information about original nanomaterial samples which would be reported via a Material file.

The transformation of the original material into the prepared sample(s) corresponds to one or more protocols (with corresponding protocol names reported in “Protocol REF” columns), associated with corresponding protocol “parameters” (reported in “Parameter Value [*parameter name*]” columns), and “factors” (reported in “Factor Value [*factor name*]” columns). The concept of “parameters” refers to “variables that are kept constant in an assay experiment”, whilst the concept of “factors” refers to “variables that are changed for studying their effects on the measured endpoint” [17]. If the assay is biological (e.g., an in vitro cytotoxicity assay), the originally sourced biological material is considered the original material, with its identifier reported in the “Source Name” column, from which a sample is prepared for testing in an assay and the originally sourced nanomaterial is considered a “factor”, since the effect of adding this nanomaterial to the biological sample being prepared for evaluation is studied: the corresponding Material file identifier (“Material Source Name”) is reported in an appropriate “Factor Value [*factor name*]” column (e.g., “Factor Value [nanomaterial]). If the assay measures nanomaterial physicochemical parameters (e.g., size by dynamic light scattering, zeta potential), the originally sourced nanomaterial sample is considered the original material, i.e., the “Material Source Name” is reported in the Study file “Source Name” column. It follows that different Study files must be created for samples prepared for biological or physicochemical assays.

#### Assay file

An Assay file [39] links (a subset of) the prepared samples described in a given Study file to the experimental measurements, of a given type, obtained in a given assay. Each Assay file row corresponds to a given sample, with the “Sample Name” identifier defined in the corresponding Study file being reported in the Assay file “Sample Name” column. Additional columns (“Protocol REF”, “Assay Name”, “Parameter Value [*parameter name*]”, “Factor Value [*factor name*]”) in the Assay file identify the assay protocol performed and experimental details associated with the production of a given (set of) data point(s) obtained from that assay for a given sample. (Here, the concepts of “parameters” and “factors” are as defined above for the Study file, although Assay file “parameters” are specific to Assay file protocols and one may choose to report “factors” in the Assay file if they are applicable to the assay procedure used to generate data points for a given prepared sample [17,39].) The corresponding data points are presented in “Measurement Value [*statistic(measurement name*)]” columns, e.g., “Measurement Value [z-average(hydrodynamic diameter)]” for an Assay file describing dynamic light scattering (DLS) size measurements [41–42].

#### External files

“External” files [17,36], presenting additional information associated with the original nanomaterial samples or assay measurements, can be linked to the appropriate Material and Assay file respectively via additional columns and may also be included within the ISA-TAB-Nano dataset.

#### Support for (meta)data standardisation

The ISA-TAB-Nano specification promotes standardised reporting of (meta)data in the following ways. (1) It defines a certain number of fixed fields (rows in the Investigation file, or columns in the remaining file types). (2) It describes a syntax for adding additional fields of a given type, e.g., “Parameter Value [*parameter name*]” and “Factor Value [*factor name*]”. (3) It supports links between terms added by the end user (e.g., a *parameter name* or the unit for a “Measurement Value [*statistic(measurement name)*]” column entry) and standardised definitions retrieved from ontologies. (An excellent introduction to ontologies can be found in the recent articles of Thomas et al. [2,11] along with an overview of a highly relevant example: the NanoParticle Ontology (NPO) [2].) (4) It supports links to standardised protocol documentation, for sample preparation or assay measurements, for protocol names reported in “Protocol REF” columns in a Study or Assay file. (The ontologies to which various terms are linked are defined using fields in the Investigation file, which also provides links between protocol names and standardised documentation.)

As well as providing some pre-defined fields and stipulating a specific syntax for adding fields of a specific type (e.g., “Factor Value [*factor name*]”), miscellaneous additional fields can be created via adding new “Comment [*name of (meta)data item*]” fields if no appropriate alternative exists.

### 2 Challenges associated with the generic ISA-TAB-Nano Specification which were addressed in the current Work

Table 1 presents some key challenges associated with the use of the generic ISA-TAB-Nano specification (version 1.2), especially when used to collect data from the published literature, and which were addressed in the work reported in the current article. An in-depth explanation of these challenges, along with a detailed discussion of the manner in which they were addressed via the use of the templates and business rules summarised in sections 3 and 4, respectively, is provided in Supporting Information File 1. It should be noted that not all of these challenges are specific to ISA-TAB-Nano, i.e., some of them might be encountered when collecting data from the literature using other formats, and by no means are all of these challenges specific to collection of data from the published literature, i.e., some of them might be encountered when trying to report primary experimental data according to the generic ISA-TAB-Nano specification. It should also be noted that not all of these challenges are necessarily within the scope of the generic ISA-TAB-Nano specification to resolve, e.g., the definition of appropriate minimum information criteria. The need to address these challenges informed the design of the templates discussed in section 3 and the accompanying business rules, summarised in section 4 and presented in full in Supporting Information File 1, which were applied for the purpose of data collection from the nanotoxicology literature within the NanoPUZZLES EU project. It should be noted that no claim is made that all of these challenges are perfectly addressed via use of the resources presented in the current publication. The strengths and weaknesses of the manner in which these issues are addressed via the templates and business rules developed within NanoPUZZLES are discussed in the context of the detailed explanation of these challenges, which is presented in Supporting Information File 1. In addition, some of these challenges are returned to in the context of considering notable limitations of the resources developed within NanoPUZZLES. These notable limitations are summarised in section 7 and discussed in detail in Supporting Information File 1.

**Table 1:** Summary of challenges with the generic ISA-TAB-Nano specification which were addressed in the current work.

no.	challenge	Applicable, in principle, to any format rather than being specific to ISA-TAB or ISA-TAB-Nano?	Applicable to ISA-TAB?	Applicable to ISA-TAB-Nano?

1	Standardised reporting of stepwise sample preparation needs to be established.	×	×	×
2	Ambiguity exists regarding where different kinds of information should be recorded.	—	×	×
3	Standardised recording of imprecisely reported experimental variables and measurements is required.	×	×	×
4	Ambiguity exists regarding the creation of “Comment […]” fields.	×	×	×
5	Statistical terms need to be clearly defined.	×	×^a^	×^a^
6	Ambiguity exists regarding how to link to terms from ontologies.	—	—	×
7	Ambiguity exists regarding whether or not “Parameter Value” or “Factor Value” column entries must be constant or not constant respectively.	—	×	×
8	Linking to images reported in publications is challenging.	×	×	×
9	Standardised reporting of multiple component “characteristics”, “factors”, and “parameters” (e.g. mixtures) needs to be established.	—	×	×
10	A standardised means of linking multiple “external” files to a given Material file is required.	—	—	×
11	Greater clarity regarding the existence of “unused” factors, parameters and measurement names in the Investigation file is required.	—	×^a^	×
12	A standardised approach for dealing with “non-applicable” metadata is required.	×	×	×
13	The concept of an “investigation” should be more tightly defined for the purpose of collecting data from the literature.	—	—	×
14	Clearly defined minimum information criteria are required.	×	×	×

^a^It should be noted that ISA-TAB is not designed to record experimental measurements in Assay files, i.e., the “Measurement Value [*statistic*(*measurement name*)]” Assay file columns and the corresponding Investigation file “Study Assay Measurement Name” field are an ISA-TAB-Nano extension [17,37,39]. However, regarding the issue of clearly defining statistical terms (challenge no. 5), ISA-TAB datasets may include “external” data files (i.e., “external” to the basic Investigation, Study and Assay file types) such as “data matrix” files which may include statistical terms such as “p-value” [36,43]. Standardisation of statistical terms may be achieved via using terms from the STATistics Ontology (STATO) [44]. The challenge noted here (challenge no. 5) regarding clearly defining statistical terms concerns how to appropriately create links to ontologies for these terms in ISA-TAB-Nano datasets.

### 3 NanoPUZZLES data collection templates

#### General overview of templates

These templates were developed within the NanoPUZZLES project [33] and were specifically designed for collection of nanotoxicology data from the literature to support analysis of trends and the development of data driven computational models such as nano-QSARs. These templates are available from the myExperiment online repository [45–46]: file entry “NanoPUZZLES ISA-TAB-Nano Templates” [47]. Version 3 of this file entry corresponds to the version of the templates referred to in the current publication and any corrections and/or extensions of these templates will also be made publicly available via future versions of this file entry.

The motivation for employing non-generic templates, designed to record specific kinds of (meta)data of interest to specific researchers, as opposed to generic templates that merely indicate the kinds of fields which the four ISA-TAB-Nano file types (Investigation, Study, Assay, Material) can contain, is that specific files with specific fields would need to be created at the point of data collection in any case but creating these specific files “on-the-fly” (i.e., at the point of data collection) is problematic. For example, a generic Assay file template would only indicate that certain, unspecified, experimental variables and endpoint values should be recorded using “Parameter Value […]” (or other column type such as “Factor Value […]”) and “Measurement Value […]” columns, respectively. However, when collecting certain kinds of data obtained with a given assay, a specific Assay file with specific “Measurement Value […]” and “Parameter Value […]”columns (or other column types such as “Factor Value […]”) would need to be created to record the (meta)data of interest. Indeed, the Investigation file is designed to associate a given “Study Assay Measurement Type” (e.g., size) and “Study Assay Technology Type” (e.g., dynamic light scattering) with a given “Study Assay File Name”. Hence, specific templates (such as those developed in the current work) serve two important purposes: (a) they avoid the end user having to decide which specific fields, of a given type, should be created to record specific items of (meta)data; (b) they communicate to the end user which items of (meta)data should be reported in the dataset, i.e., they effectively define minimum information criteria. However, in case the specific templates do not capture all the experimental (meta)data of interest to a given end user of the dataset, it is important to recognise that the templates may be updated with new fields (in existing templates) or additional specific templates may be created.

The templates developed in the current work were adapted from generic Excel templates made available by the ISA-TAB-Nano developers [23]. The templates presented in this publication are designed to be compatible with version 1.2 of the ISA-TAB-Nano specification [23]. The generic templates were adapted as follows.

1. Predefined “Comment […]” fields were added to the Investigation file template for recording additional important metadata, e.g., “Comment [GLP]” for recording whether or not the corresponding studies were carried out according to Good Laboratory Practice [27,48].

2. Two specific Study file templates were created for sample preparation prior to physiochemical or cell based in vitro assays. (A Study file for sample preparation prior to in vivo assays was under development at the time of writing.)

3. Specific Assay file templates were created for (a) different kinds of physiochemical measurements and, in some cases, (b) for specific assays which might be employed to make those measurements. In some cases, where scenario (b) was not applicable, generic “Measurement Value [*statistic*(*measurement name*)]” columns were created with the *statistic* and/or *measurement name* presented as a generic “[TO DO: ….]” label: these labels should be replaced, as required, with specific *statistic* and *measurement name* values during data collection (as documented in the templates) or columns with these generic headings should be deleted if not applicable. For example, an Assay file template was designed for recording size measurements from a non-predetermined assay type (“a_InvID_PC_size_Method.xls”) in addition to some Assay file templates for recording size measurements obtained using specific assay types - such as dynamic light scattering (DLS) (“a_InvID_PC_size_DLS.xls”) [41–42]. The former template (“a_InvID_PC_size_Method.xls”) includes the column “Measurement Value [[TO DO: appropriate average]([TO DO: appropriate size measurement])]”: this would be updated to “Measurement Value [mean of the number distribution(diameter)]”, to give but one possible example, during dataset creation. The latter template (“a_InvID_PC_size_DLS.xls”) includes the columns “Measurement Value [z-average (hydrodynamic diameter)]” and “Measurement Value [polydispersity index]”.

4. Specific Assay file templates were created for recording toxicity data for endpoints that were prioritised within the NanoPUZZLES project.

5. Predefined “Characteristics […]”, “Factor Value […]” and “Parameter Value […]” columns were added to these Study and Assay file templates based upon consideration of which experimental variables were expected to affect the associated assay measurements. For example, the Study template for cell based in vitro studies (“s_InvID_InVitro.CB.xls”) includes the predefined columns “Characteristics [cell type {EFO:http://www.ebi.ac.uk/efo/EFO_0000324}]” and “Factor Value [exposure medium]”.

6. Predefined “Characteristics […]” columns were added to the Material file template for recording important chemical composition information, beyond that specified in the generic templates, along with nominal/vendor supplied values of various other physicochemical parameters, e.g., “Characteristics [Product impurities found {MEDDRA:http://purl.bioontology.org/ontology/MDR/10069178}]”, “Characteristics [Major crystalline phase]” and “Characteristics [average size]”.

7. Predefined “Comment […]” columns were added to the Material, Study and Assay file templates for recording key metadata that could (a) assist in interpreting the results or (b) allow the quality of the results to be assessed. For example, the template “a_InvID_PC_size_TEM.xls” for recording size by transmission electron microscopy (TEM) contains the columns “Comment [primary particle measurements]” and “Comment [size: from graph]” to address requirements of type (a) and (b) respectively. The “Comment [primary particle measurements]” column was designed to report whether or not the size measurements obtained were explicitly stated, in the publication from which they were extracted, to have been made for the primary particles: in principle, TEM might be used to provide information about agglomerates, aggregates or primary (individual) particles for a given prepared sample [49–50]. The “Comment [size: from graph]” column was predicated on the assumption that data extracted from graphs (which are not uncommon when collecting data from the literature) are less reliable (i.e. more prone to transcription errors) than data extracted from tables or text.

8. For some fields, drop-down lists with possible field entries were created using the “Data Validation” option in Excel 2010.

9. The fields were colour coded to indicate those fields which were judged to be essential (green), desirable (yellow) or not important for the purposes of the NanoPUZZLES project (red).

10. Some fields (e.g., the Material file “Material Design Rationale” column) which were not considered important for the purposes of the NanoPUZZLES project were simply deleted.

11. Detailed comments were added (via the Excel 2010 “Review” tab) describing how different predefined fields should be populated during data collection.

12. The fields in the Investigation template (“i_InvID.xls”) were populated insofar as possible prior to data collection. This included specifying predefined “factors” and “parameters” (c.f. other templates) and defining a set of ontologies from which terms should (preferentially) be obtained during data collection.

13. Some of the fields in the templates were populated with *indicated* values where appropriate. In some cases, these indications might actually be literally entered as values for the corresponding field entries, e.g., “size determination by DLS” entered in the first row of the “Protocol REF” column in the “a_InvID_PC_size_DLS.xls” template. However, in other cases, the suggested entries should not be entered literally, e.g., “size determination by <Assay technology type>” entered in the first row of the “Protocol REF” column in the “a_InvID_PC_size_Method.xls” template, where “<Assay technology type>” would be replaced with the name of the relevant method, such as “environmental scanning electron microscopy” [51–52] for the Assay file (“a_TOY.article_PC_size_ESEM.xls”) in the “Toy Dataset” (see section 6) derived from the template “a_InvID_PC_size_Method.xls”.

14. NanoPUZZLES specific naming conventions were established (as suggestions, rather than business rules) for creating files based on these templates. For example, “InvID” denotes “Investigation Identifier” and “Method” denotes an assay measurement technique such as dynamic light scattering (DLS).

15. A new “ImageLink” template was created (“ImageLink_NUMBER_for_InvID.xls”) for linking to images reported in publications which are not associated with a single file that can be redistributed as part of a dataset or uniform resource identifier (URI). The use of this template is defined by NanoPUZZLES business rule no. 18 (see section 4 and Supporting Information File 1).

#### Identification of important experimental variables and characterisation data

The experimental variables (for both toxicological and physicochemical assays) and types of physicochemical characterisation data which the templates were designed to capture were based upon considering the well-known MINChar Initiative Parameters List [53], the provisional recommendations developed within the NanoSafety Cluster Databases Working Group [26], other resources developed within the context of the NanoSafety Cluster projects PreNanoTox [54] and MARINA [55] as well as discussions with nanotoxicology researchers and consideration of the published literature regarding toxicologically significant physicochemical characterisation parameters (for nanomaterials) and experimental variables which could significantly affect toxicological or physicochemical measurements [10,12,49,56–63]. However, no claim is made that the templates developed to date within the NanoPUZZLES project would capture all of the experimental variables or relevant characterisation information indicated by the cited proposals or otherwise recognised as important in the nanotoxicology community.

#### Physicochemical characterisation data captured by the templates

The categories of physicochemical information these templates were designed to capture, along with the corresponding Material and/or Assay file templates, are summarised in Table 2. In keeping with the generic ISA-TAB-Nano specification (version 1.2) [64], information which could be recorded using an Assay file template (“a_.....xls”) should only be recorded using the Material file template (“m_MaterialSourceName.xls”) if its value was nominal or vendor supplied.

**Table 2:** Categories of physicochemical information which the NanoPUZZLES ISA-TAB-Nano templates were designed to capture.

category	template(s)	comments

chemical composition (including surface composition, purity and levels of impurities)	“m_MaterialSourceName.xls”	Only chemical composition information associated with the original / vendor supplied nanomaterial should be reported here, i.e., not adsorption data (see below).
crystal structure/ crystallinity	“m_MaterialSourceName.xls”; “a_InvID_PC_crystallinity_Method.xls”	—
shape	“m_MaterialSourceName.xls”; “a_InvID_PC_shape_Method.xls”	Both qualitative descriptions of shape or “aspect ratio” data [60] can be recorded.
particle size/ size distribution	“m_MaterialSourceName.xls”; “a_InvID_PC_size_Method.xls”; “a_InvID_PC_size_DLS.xls”; “a_InvID_PC_size_TEM.xls”	Dynamic light scattering (DLS) [41] or transmission electron microscopy (TEM) [65–66] measurements are captured using the indicated Assay file templates. Otherwise, unless size values are nominal/vendor supplied, size measurements are captured via the generic Assay file template.
surface area	“m_MaterialSourceName.xls”; “a_InvID_PC_surface area_Method.xls”	This was designed to record “specific surface area” values, i.e., surface area per unit mass [58].
surface charge/ zeta potential	“m_MaterialSourceName.xls”; “a_InvID_PC_zetapotential_Method.xls”	Zeta potential is commonly used as a proxy for surface charge [58].
adsorption	“a_InvID_PC_adsorption_Method.xls”	This was designed to record “adsorption constants” [57] and (equilibrium) adsorption percentages [67] for specific small molecule / macromolecular “probe” species.
reactivity	“a_InvID_PC_reactivity.rateofchange_of.X_SeparationTechnique_Method.xls”	The design of this template reflects the fact that, for some reactivity assays, the analysed species needs to be removed prior to making measurements [68].
dissolution	(1) “a_InvID_PC_dissolution.conc_of.X_SeparationTechnique_Method.xls” ; (2) “a_InvID_PC_dissolution.fraction-dissolved_SeparationTechnique_Method.xls”; (3) “a_InvID_PC_dissolution.rate_of.X_SeparationTechnique_Method.xls”	The design of these templates reflects the fact that a number of different kinds of dissolution measurement may be made for inorganic nanoparticles: (1) the (time dependent) concentrations of various species released by dissolution [67,69] (which may be a redox process [69]); (2) the (time dependent) percentage of original nanoparticles dissolved [70]; (3) the (time dependent) dissolution rate [71]. The design of these templates further reflects the fact that dissolution assay protocols typically employ a separation step to isolate the analysed species [61].
molecular solubility	“a_InvID_PC_solubility_Method.xls”	In the current context, the Chemical Methods Ontology definition of “solubility” [72] was used: “the concentration of a solute in a saturated solution”. This Assay template was specifically designed for recording molecular “solubility” measurements, e.g., the solubility of fullerene nanoparticles [73].
agglomeration/ aggregation	“a_InvID_PC_AAN_BETapproach.xls”	This template was designed for recording the “average agglomeration number” derived from BET gas adsorption data, size measurements and particle density values [58,74]. However, it should be noted that recording of size information obtained under different experimental conditions (using the Assay file templates noted above) may also convey information about the agglomeration state [58]. In addition, a number of physicochemical Assay files (e.g. “a_InvID_PC_size_Method.xls”) contain “Comment […]” columns (e.g., “Comment[primary particle measurements]”) designed to record whether or not the reported data are noted to refer to the primary particles (as opposed to agglomerates and/or aggregates) by the authors of the reference from which the data were extracted.
hydrophobicity	“m_MaterialSourceName.xls”; “a_InvID_PC_logP_Method.xls”	—

These categories of physicochemical information correspond to all of the kinds of physicochemical information highlighted as being important in the MINChar Initiative Parameters List [53], with the context dependence stressed by this initiative being (partially) captured via recording sample conditions using “Factor Value […]” columns in the physicochemical Study file template (“s_InvID_PC.xls”), e.g., “Factor Value [medium]”.

In order to construct these templates, careful consideration was required of exactly how to record different kinds of physicochemical information highlighted as being important. Firstly, this required consideration of which measurements might correspond to different kinds of physicochemical information; the “minimum” characterisation parameters reported in various proposals [12,53] are sometimes quite broadly defined, e.g., “Surface Chemistry, including reactivity, hydrophobicity” [53]. Secondly, this required consideration of which corresponding Material file “Characteristics […]” and/or Assay file “Measurement Value […]” columns needed to be defined - as well as, in some cases, which “Parameter Value […]” columns needed to be defined, e.g., “Parameter Value [analyte role]” (i.e., the dissolved species being measured) for dissolution Assay file templates. No claim is made that the templates developed to date within the NanoPUZZLES project would capture all relevant measurements which might be associated with a given category of physicochemical information listed in Table 2.

#### Experimental variables captured by the templates

The experimental variables associated with sample preparation prior to applying assay protocols for (1) physicochemical measurements (see above) or (2) cell based in vitro toxicological assays are principally described via “Factor Value […]” columns in two Study file templates: (1) “s_InvID_PC.xls”, (2) “s_InvID_InVitro.CB.xls”.

For physicochemical studies, these “Factor Value […]” columns record the values of experimental variables associated with the preparation of a nanomaterial sample prior to application of an assay protocol, e.g., “Factor Value [physical state]” (for recording whether or not the sample was prepared as a suspension or a powder), “Factor Value [medium]” (for recording the suspension medium, i.e., not applicable if the “physical state” is a powder), “Factor Value [Sonication]” (for recording whether or not the sample was sonicated [49]).

For cell-based in vitro studies, these “Factor Value […]” columns record the values of experimental variables associated with preparation of the composite sample being tested, i.e., the nanomaterial suspension and the biological component on which the effect of the nanomaterial will be evaluated. Hence, they are designed to capture different kinds of experimental variables: (1) those which are relevant to preparation of the biological sample prior to adding the nanomaterial, e.g., the “Factor Value [culture medium glucose supplement]” in “s_InvID_InVitro.CB.xls” designed to record whether or not the cells were grown in glucose containing “culture medium”, which may significantly affect the observed toxicity in some in vitro assays [56]; (2) those which are relevant to the preparation of the nanomaterial sample applied to the biological sample, e.g., “Factor Value [exposure medium]” and “Factor Value [Sonication]” for capturing the “exposure medium” for an in vitro (cell-based) study (otherwise known as the “exposure media” [75–76], i.e., the liquid mixture via which the tested chemical – a nanomaterial in the current context - reaches the cells) and whether or not sonication was applied to the tested nanomaterial suspension respectively; (3) those which are relevant to the combined sample to which the assay protocol is applied, e.g., “Factor Value [cells Exposure Duration]”.

Capturing of the experimental conditions under which corresponding physicochemical characterisation and toxicity data were generated is important to assess whether or not characterisation was performed under biologically relevant conditions [77]. For example, whether or not a given size measurement was performed in the same suspension medium used for an in vitro (cell-based) study might be determined via comparing the “Factor Value [medium]” and “Factor Value [exposure medium]” entries in the physicochemical and in vitro (cell-based) Study files, respectively. However, details regarding possible suspension medium additives – such as serum and dispersant aids [78] – would need to be compared with each other by comparing the values in additional “Factor Value […]” fields.

In addition, for the “s_InvID_InVitro.CB.xls” Study file template, “Characteristics […]” columns associated with the “Source Name” column (i.e., positioned after the “Source Name” column but before the “Sample Name” column) are used to describe experimental variables which are inherent to the biological specimen: "cell type”, “cell line”, “organism” and “strain”, as defined in the Experimental Factor Ontology (EFO) [79–80].

Experimental variables specifically associated with assay protocols are recorded in Assay files, principally using “Parameter Value […]” columns, e.g., “Parameter Value [Instrument]”, “Parameter Value [negative control]”.

It should be noted that the manner in which some of these experimental variables are captured via these templates might be carried out differently by other researchers and may deviate from the expectations of the generic ISA-TAB(-Nano) specification [17,23,36]. Some of the “Factor Value […]” columns (e.g., “Factor Value [physical state]” or “Factor Value [final cell density]” in “s_InvID_PC.xls” and “s_InvID_InVitro.CB.xls” respectively) might be considered to refer to characteristics of the prepared sample. Hence, these kinds of variables might elsewhere be recorded using “Characteristics […]” columns associated with the “Sample Name” column, i.e., positioned after the “Sample Name” column [36]. Other variables recorded via “Factor Value […]” columns (e.g., “Factor Value [Sonication Duration]”) might be kept constant in some experiments [81], hence could be considered protocol parameters which would be recorded using “Parameter Value […]” columns [17]. However, the use of “Factor Value […]” columns to record these latter variables was deemed appropriate to account for scenarios in which these variables (e.g., sonication duration) were varied to assess their effect on assay measurements [49]. The fact that certain kinds of variables might be considered, in keeping with the generic ISA-TAB-Nano specification [17] discussed in section 1, “parameters” in one set of experiments and “factors” in another depending upon whether or not they were kept constant or varied to study their effects on the assay measurement values does not lend itself to consistently organising these experimental variables in predefined template columns as developed in the current work.

The potential ambiguity associated with how to record different experimental variables can be illustrated by considering differences between the NanoPUZZLES ISA-TAB-Nano [47] and ToxBank ISA-TAB templates [82–83]: (1) the NanoPUZZLES Study file template “s_InvID_InVitro.CB.xls” contains the column “Factor Value [exposure medium]” for describing the suspension medium via which a tested nanomaterial is applied to the cells in an in vitro study, whereas the ToxBank Study file template “studySample.xml” contains the column "Characteristics[vehicle]" for describing the medium used to dilute a tested compound in an in vitro, in vivo or ex vivo study; (2) the NanoPUZZLES Assay file templates treat the identity of assay controls as “Parameter Value […]” entries (e.g., “Parameter Value [negative control]”), whereas the ToxBank Study file template uses a “Characteristics […]” column ("Characteristics[control]") to assign negative or positive control status to different samples.

#### Toxicity data captured by the templates

Assay file templates were developed to capture toxicity data associated with two toxicological endpoints which were initially prioritised within the NanoPUZZLES project: cytotoxicity (“a_InvID_cytotoxicity.cell-viability_Method.xls”, “a_InvID_cytotoxicity.sub-lethal_Method.xls”) and genotoxicity (“a_InvID_genotoxicity_Method.xls”). Cytotoxicity and genotoxicity are amongst the endpoints which are frequently considered when evaluating metal oxide nanoparticles in cell-based in vitro assays [4,84]. A number of nano-QSAR models have been developed for cytotoxicity [13,85–91] and some models have also been developed for nanomaterial genotoxicity [9,92–93].

The genotoxicity Assay file template (“a_InvID_genotoxicity_Method.xls”) was designed to capture the most important outputs from different kinds of genotoxicity tests. Specifically, the “Parameter Value [Biomarker]” was designed to record the, test specific, biomarker whose increase relative to control values (“Measurement Value [mean(increase in biomarker level)]”) would be determined for nanomaterial exposed samples. For example, “Parameter Value [Biomarker]” might report “micronuclei” or “number of revertants” if the method employed was the micronucleus test [94] or Ames test [95–96] respectively.

Since the results obtained for different sample preparation conditions (e.g., different tested concentrations) are usually used to derive an overall genotoxicity study call (i.e., “positive”, “negative” or “equivocal”) [94,96], a corresponding “Measurement Value [study call]” was added. Values in this latter column should be associated with “derived sample” identifiers as introduced in NanoPUZZLES business rule no. 10 (see section 4 and Supporting Information File 1 for an in-depth explanation).

The lethal cytotoxicity Assay file template (“a_InvID_cytotoxicity.cell-viability_Method.xls”) was designed to record data corresponding to a reduction in cell “viability” (typically interpreted as an increase in “cell death”) obtained from cell based in vitro assays such as MTT, MTS, LDH, and colony forming unit (CFU) counting [97–99]. The “percent cytotoxicity” columns (“Measurement Value [mean(percent cytotoxicity)]”, “Measurement Value [standard deviation(percent cytotoxicity)]”) are designed to record the “percent cytotoxicity” (a measure of cell death relative to controls equal to 100 – “percent viability”) [100] associated with specific sample preparations, i.e., a specific value for the administered concentration or dose [101]. Other “Measurement Value […]” columns were designed to record measures of cytotoxicity derived from dose (or concentration) response relationships: the lowest observed effect level (LOEL) [102] (used, in the current work, to denote the lowest concentration/dose at which significant cell death relative to controls is observed), the LC_50_ [103] and LD_50_ [104], i.e., the concentration and dose, respectively, which, in the current context, kills 50% of the treated cells relative to controls. Values in these latter columns should be associated with “derived sample” identifiers as introduced in NanoPUZZLES business rule no. 10 (see section 4 and Supporting Information File 1 for an in-depth explanation).

The sub-lethal cytotoxicity Assay file template (“a_InvID_cytotoxicity.sub-lethal_Method.xls”) was designed to record data from cell based in vitro assays designed to detect sub-lethal phenomena which might be quantified in terms of changes in key biomarkers. For example, oxidative stress and inflammation might be detected via measuring the level of glutathione or various cytokine biomarkers respectively [97]. (These sub-lethal phenomena would not be considered “cytotoxicity” by all researchers [84].) The manner in which this template was designed to capture sub-lethal cytotoxicity data is similar to the design of the genotoxicity Assay file template discussed above: the “Parameter Value [Biomarker]” column entries would state, for example, “glutathione” (depending upon the assay), with “Measurement Value […]” columns recording the “increase in biomarker level” (relative to control) as well as the LOEL [102] if this is reported. Values in this latter column should be associated with “derived sample” identifiers as introduced in NanoPUZZLES business rule no. 10 (see section 4 and Supporting Information File 1 for an in-depth explanation).

### 4 NanoPUZZLES business rules

Within the NanoPUZZLES project [33], a number of project specific business rules were created for the purpose of specifying how the ISA-TAB-Nano templates described in section 3 should be populated with data from literature sources. As noted in section 2, and fully explained in Supporting Information File 1, some of these business rules were specifically designed to address challenges associated with the generic ISA-TAB-Nano specification. A summary of these business rules is provided in Table 3. Supporting Information File 1 presents detailed explanations of how these business rules should be applied and, where appropriate, considers their strengths and weaknesses compared to possible alternatives which might be applied in future work.

**Table 3:** Summary of the NanoPUZZLES business rules.

business rule no.	short description

1	A new “investigation” (corresponding to a new dataset comprising a single Investigation file, a set of Study, Assay and Material files and any “external” files if applicable) should be created for each reference (e.g., journal article), unless that reference specifically states that additional information regarding experiments on the same original nanomaterial samples was reported in another reference.
2	The “Factor Value […]” columns in the Study file refer to those values which are applicable to the sample prepared immediately prior to application of an assay protocol.
3	If the entry for a “Characteristics […]”, “Factor Value […]” or “Parameter Value […]” column corresponds to multiple components (e.g., mixtures), record this as a semicolon (“;”) delimited list of the separate components.
4	If the entry for a “Characteristics […]”, “Factor Value […]” or “Parameter Value […]” column corresponds to multiple components, record the entries in corresponding columns as a semicolon (“;”) delimited list with the entries in the corresponding order.
5	Any intrinsic chemical composition information associated with a nanomaterial sample (as originally sourced) should be recorded using a Material file even if it is determined/confirmed using assay measurements reported in the publication from which the data were extracted.
6	Any suspension medium associated with the nanomaterial sample (as originally sourced) should only be described using a Material file “Material Description” column.
7	Any impurities should be described using entries in the relevant Material file “Characteristics [….]” columns.
8	Any original nanomaterial components, which are neither a suspension medium nor described as “impurities” in the reference from which the data are extracted, should be described using separate rows of the Material file as per the generic ISA-TAB-Nano specification.
9	All “Sample Name” values for “true samples” should have the following form: “s_[Study Identifier]_[x]”, e.g., “s_[Study Identifier]_1”^a^
10	Assay file “Measurement Value […]” column entries which correspond to concentration-response curve statistics, or similarly derived measures, should be associated with a “derived sample” identifier rather than a “true sample” identifier.
11	Imprecisely reported experimental variables should be reported using “Factor Value [statistic(original factor name)]” columns created “on-the-fly”.
12	Imprecisely reported measurement values should be reported using “Measurement Value [statistic(measurement name)]” columns created “on-the-fly”.
13	“Comment […]” columns (rows) can be added without restriction to a Study, Assay, Material (Investigation) file as long as they are appropriately positioned and as long as each new “Comment […]” column (row) has a unique name for a given file.
14	All “statistic” names must be entered in the corresponding Investigation file template “Comment [Statistic name]” row.
15	When linking to terms from ontologies, the “preferred name” should be selected and the full ID entered in the corresponding “Term Accession Number” field.
16	“Factor Value […]” column entries are allowed to be constant.
17	Only “Parameter Value […]” column entries associated with a given “Protocol REF” column entry in a Study or Assay file need to be constant.
18	Images should be linked to assay measurements using a new “ImageLink” file type, if the generic ISA-TAB-Nano approach cannot be applied.
19	Any nanomaterial structure representation files, which are not associated with specific Assay file “Measurement Value […]” entries, should be linked to the corresponding Material file using ZIP archives specified in the appropriate “Material Data File” column entry.
20	Empty “Factor Value […]”, “Parameter Value […]” or “Measurement Value […]” columns in Study or Assay files can be deleted without having to update the corresponding Investigation file “Study Protocol Parameters Name”, “Study Factor Name”, or “Study Assay Measurement Name” fields.
21	Non-applicable columns should be populated with “N/A” where this conveys information.
22	“Measurement Value [statistic(measurement name)]” columns in the templates which use a label of the form “[TO DO:…]” for the statistic or measurement name must either be updated, based on the kind of statistic and/or measurement name indicated by the label(s), or deleted.

^a^Here, the “[Study Identifier]” [37] is unique to the corresponding Study file and “[x]” denotes a numeric value which is specific to a given “true sample”, meaning a prepared sample corresponding to a specific set of experimental conditions, in contrast to the “derived sample” concept introduced in NanoPUZZLES business rule no. 10.

These new rules were applied in addition to the rules which are part of the generic ISA-TAB-Nano specification as of version 1.2 [17,23,36–40]. (The new rules took precedence over the generic specification in case of conflicts.) It should also be remembered that additional guidance on creating ISA-TAB-Nano datasets using these templates is provided in section 3 and that guidance on populating individual fields is provided in the Excel-created comments linked to specific column titles. Finally, in keeping with the generic specification, the Investigation file and all corresponding files (Study, Assay and Material files along with all external files when applicable), for a single dataset, were added to a single, flat compressed ZIP archive (see section 5).

### 5 NanoPUZZLES Python program to facilitate computational analysis and database submission

Excel-based ISA-TAB-Nano templates are presented in this publication and elsewhere [17,23]. However, ISA-TAB-Nano files (Investigation, Study, Assay, Material) are commonly implemented in tab-delimited text format [105], reflecting the fact that ISA-TAB-Nano is an extension of ISA-TAB and ISA-TAB is intended to be implemented using tab-delimited text files (Investigation, Study, Assay) [36]. The authors of the current publication are unaware of any software specifically designed for parsing ISA-TAB datasets [22,82,106], which might be extended to parse ISA-TAB-Nano datasets, or software specifically designed for parsing ISA-TAB-Nano datasets [107–108], which does not require the key file types (Investigation, Study, Assay and, for ISA-TAB-Nano, Material) to be represented in tab-delimited text format. This includes publicly available online resources recently developed within the context of the MODERN project [107]: an ISA-TAB-Nano dataset validator and “Nanomaterial Data Management System” (“nanoDMS”) – with the latter program implementing a web-based, searchable database system which is able to, amongst other functionality, import validated ISA-TAB-Nano datasets [30,109–110].

To facilitate database submission and other computational analysis, a Python [111] program was written, within the context of the NanoPUZZLES project, to enable automated conversion of an ISA-TAB-Nano dataset prepared using Excel-based templates to a tab-delimited text version of this dataset. Specifically, this program was designed to take a flat, compressed ZIP archive (e.g., “Investigation Identifier.zip”) containing Excel (“xls”) versions of an Investigation file, plus corresponding Study, Assay and Material files, and convert this to a flat, compressed ZIP archive (e.g., “Investigation Identifier-txt.zip”) containing tab-delimited text versions of these files. Any external Excel-based “xls” files (e.g., “ImageLink” files introduced in the current work) contained in the archive will also be converted to tab-delimited text files and other external files will be transferred to the new archive without modification.

The program has four Open Source dependencies: a Python interpreter [111] along with the xlrd, xlwt [112] and unicodecsv [113] Python modules. For the purposes of code development, Python version 2.7.3, xlrd version 0.93, xlwt version 0.7.5 and unicodecsv version 0.9.4 were employed. All code was tested on a platform running Windows 7. The program does not have a graphical user interface (GUI): input is specified from the command prompt, e.g., “python xls2txtISA.NANO.archive.py –i InvestigationID.zip”. The source code and documentation are available via the “xls2txtISA.NANO.archive” project on GitHub [114]. Version 1.2 of the program is referred to in the current publication [115].

**Figure 2:**
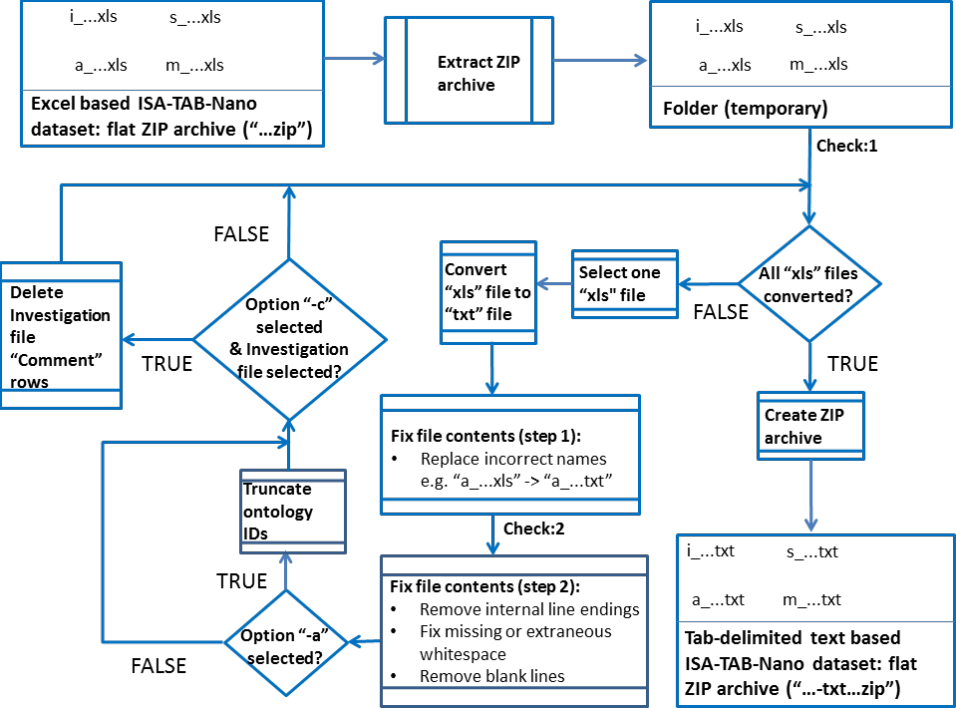
Schematic overview of the steps carried out by the Python program for converting Excel (“xls”) based ISA-TAB-Nano datasets to tab-delimited text (“txt”) based ISA-TAB-Nano datasets. For simplicity, only one Investigation, Study, Assay and Material file (and no external file such as an image) is included in this hypothetical dataset. In addition to the file processing steps summarised in this schematic, basic checks are carried out on the input: (1) there should be at least one Investigation, Study, Assay and Material file; (2) there should be no duplicate column titles in a Study, Assay or Material file other than those which are explicitly allowed by the ISA-TAB-Nano specification (e.g., “Unit”).

Figure 2 provides an overview of the functionality of the program. As part of converting from Excel-based to tab-delimited text versions of ISA-TAB-Nano files, this program carries out basic checks on the datasets (e.g., checking for the presence of at least one file of type Investigation, Study, Assay, Material) and attempts to correct for basic potential errors in the file contents (e.g., removing line endings inside field entries) which might be introduced when manually preparing ISA-TAB-Nano files using Excel templates. However, the program does not carry out any sophisticated “parsing” of the datasets, i.e., no attempt is made to interpret the data in terms of the meaning of individual fields or the contents of individual field entries. No checks are carried out on the consistency of different files. Issues such as case sensitivity, null values and special characters (beyond removing internal line endings) are not addressed. Nonetheless, by facilitating conversion to tab-delimited text format, this enables the datasets to be parsed via more sophisticated tools such as those developed for validating ISA-TAB-Nano datasets within the MODERN project [107–108].

As well as the default behaviour of this program described above, two command line options were specifically introduced to enable submission of an ISA-TAB-Nano dataset developed using these Excel templates to a database developed using the nanoDMS software [30,107,109–110]. The first option (“-a”) truncates all ontology identifiers: at the time of writing, “.” characters were not permitted by the nanoDMS system in the headers of the Material, Study or Assay files, i.e., the column heading “Characteristics [shape {NPO:http://purl.bioontology.org/ontology/npo#NPO_274}]” in the Material files generated using the default options would need to be converted to “Characteristics [shape {NPO:NPO_274}]” etc. The second option (“-c”) removes all “Comment […]” rows from the Investigation file: at the time of writing, these rows would also (indirectly) trigger errors when trying to load ISA-TAB-Nano datasets into the nanoDMS system. The output files are automatically named according to the options selected.

### 6 Toy dataset

In order to illustrate the use of all of the NanoPUZZLES template files, a “Toy Dataset” was created based upon these template files in accordance with the business rules summarised in section 4 and discussed in detail in Supporting Information File 1. It must be noted that the (meta)data contained within this “Toy Dataset” are not real, although they are based upon consideration of the nanoscience literature [4,49,51,57–58,60–61,67–68,70–71,73–74,97,116–117]. Indeed, no primary literature reports presenting data corresponding to all of the templates were identified as of the time of writing. An overview of the toy data content of this “Toy Dataset”, generated after uploading this dataset into the nanoDMS database [110], is provided below in Figure 5 and Figure 6.

**Figure 3:**
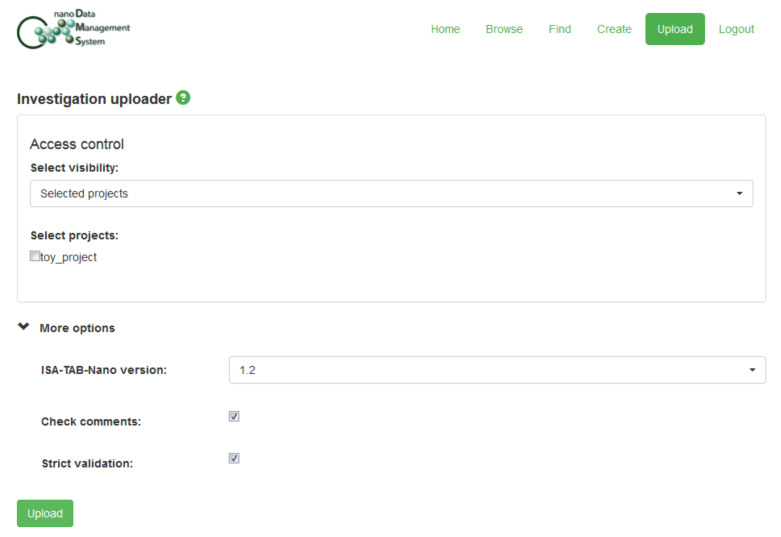
Upload options for loading the suitable version of the “Toy Dataset” (Supporting Information File 4) into the nanoDMS online database, which can be accessed via the cited web-address [110]: ontology identifiers were truncated and Investigation “Comment […]” rows deleted, using the Python program described in section 5, in order to enable this submission. Since these were not real data, the upload settings were selected such that the “Toy Dataset” was not publicly visible after uploading.

**Figure 4:**
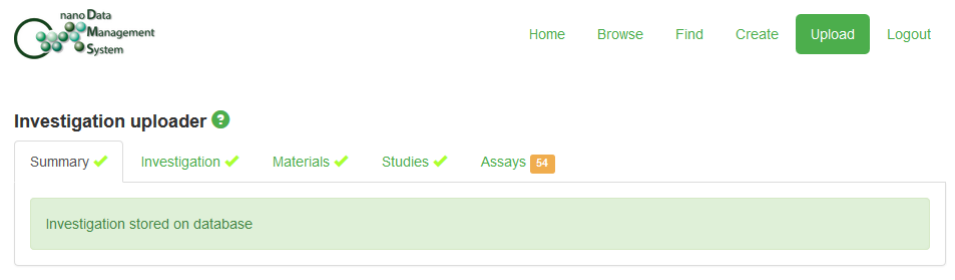
Confirmation that the “Toy Dataset” (Supporting Information File 4) was successfully uploaded: no error messages were generated by the internal ISA-TAB-Nano dataset validator and the warning messages regarding the position of the "Measurement Value [...]" and "Image File" columns reflect the addition of the “Measurement Value […]” column type to ISA-TAB-Nano, as compared to ISA-TAB, Assay files.

**Figure 5:**
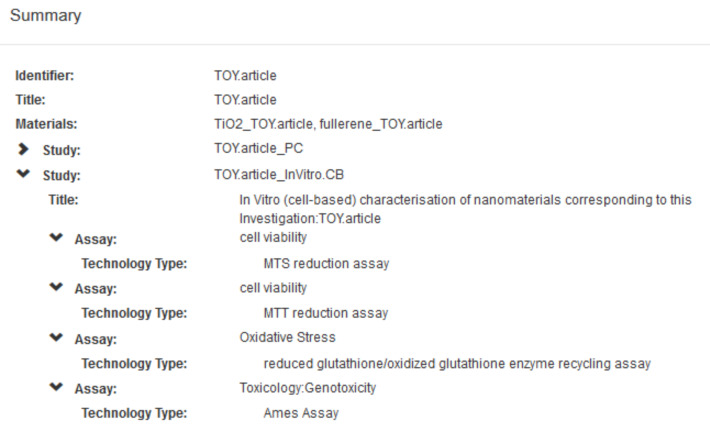
A summary of the in vitro cell-based assay toy data in the “Toy Dataset” (Supporting Information File 4) generated via the nanoDMS system. This summary can be generated via selecting the applicable dataset entry under the "Browse" menu of the nanoDMS system.

**Figure 6:**
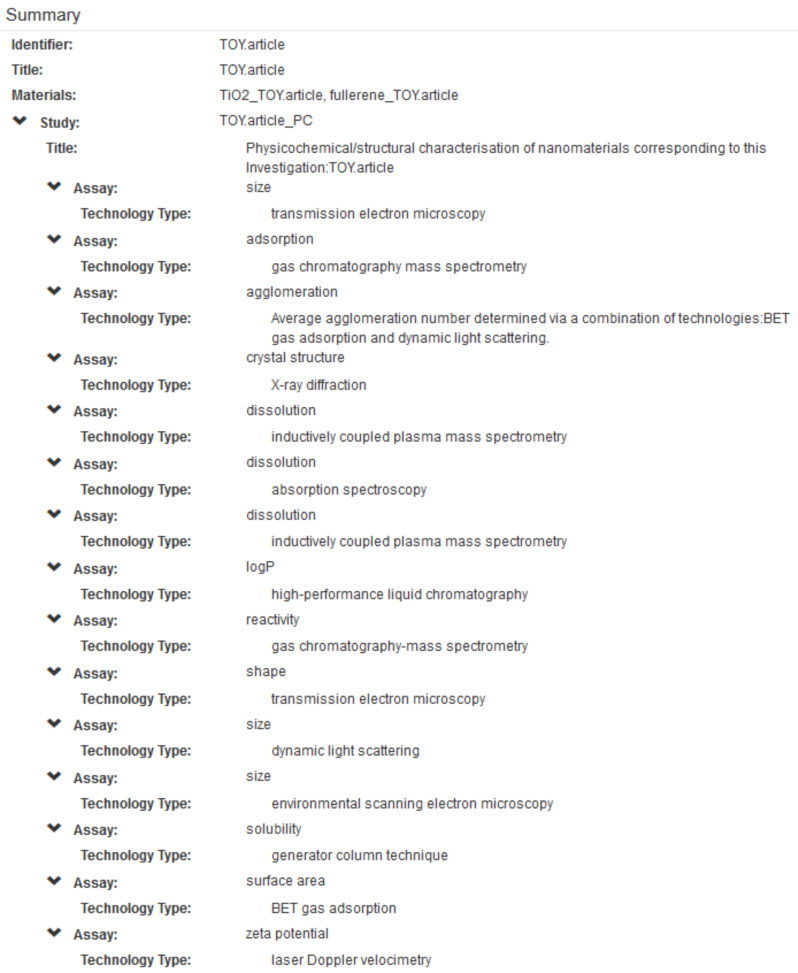
A summary of the physicochemical assay toy data recorded in the “Toy Dataset” (Supporting Information File 4), generated via the nanoDMS system as per Figure 5. This does not include the hypothetical chemical composition and nominal/vendor supplied data recorded in the Material files.

**Figure 7:**
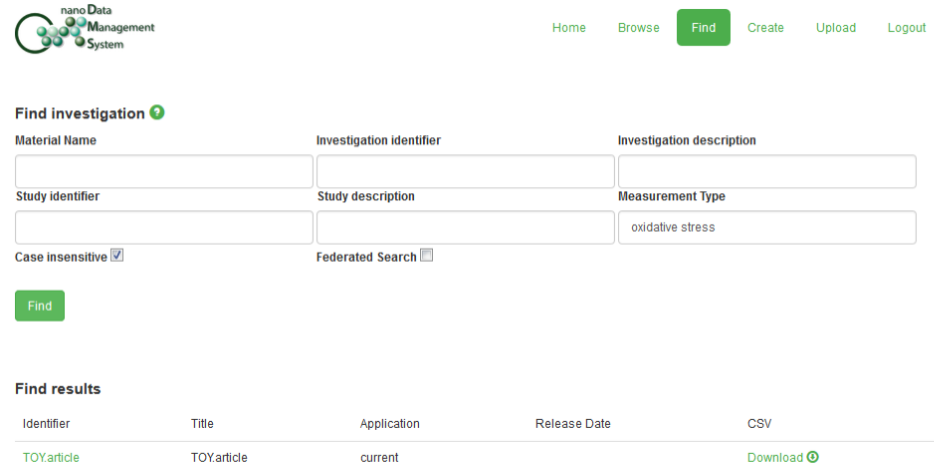
Retrieving the “Toy Dataset” (Supporting Information File 4) via searching for "oxidative stress" data in the nanoDMS system.

This “Toy Dataset” is available from the Supporting Information in three versions: Supporting Information File 2 corresponds to a flat archive containing files created using the original Excel templates and saved as “xls” files; Supporting Information File 3 is the version of this dataset created using the default options of the Python program described in section 5; Supporting Information File 4 was generated using the “-a” and “-c” flags of this software. This latter version (Supporting Information File 4) could be uploaded into the nanoDMS database [110], which is further discussed in section 7. The following figures provide an overview of the upload procedure for this dataset as well as illustrating the use of the nanoDMS system for retrieving these data: Figures 3–7.

### 7 Critical appraisal of the current work and possible future directions

#### Some notable limitations of the NanoPUZZLES templates and business rules introduced in this article

The strengths and weaknesses of the manner in which the challenges associated with the generic ISA-TAB-Nano specification (see section 2) were addressed via the templates and business rules developed within NanoPUZZLES are discussed in Supporting Information File 1. Beyond the need to address these general challenges, the specific strengths and weaknesses related to the design of the NanoPUZZLES templates (section 3) and business rules (section 4) were also discussed in section 3 and Supporting Information File 1, respectively. For example, it was noted in section 3 (under the “Experimental Variables Captured by the Templates” sub-section) that the manner in which certain experimental variables are recorded using the NanoPUZZLES templates may deviate from how other researchers would capture these metadata using ISA-TAB-Nano. Likewise, a possible alternative to the use of “derived sample” identifers (introduced in NanoPUZZLES business rule no. 10) for capturing concentration-response curve statistics, such as an LC_50_ [103], and related data is presented when discussing this business rule in Supporting Information File 1.

Table 4 summarises what are arguably the most notable remaining challenges associated with using these resources (templates and business rules) to collect nanotoxicology data from the literature. An in-depth discussion of these challenges, along with some suggestions for addressing them, is provided in Supporting Information File 1.

**Table 4:** Summary of some notable limitations of the NanoPUZZLES templates and business rules.

limitation no.	brief description

1	Standardised reporting of stepwise sample preparation is still not handled perfectly.
2	Time dependent physicochemical characterisation data may not be perfectly captured by the templates.
3	Recording of reaction rate constants and quantum yields may need revision.
4	The manner in which chemical composition information is captured via the templates may require revision.
5	There is the possibility of information loss when mapping (raw) data reported in the literature onto predefined “Measurement Value […]” columns.
6	The current templates are not best suited to capturing experimental data for all kinds of samples.
7	The business rules regarding multiple component “characteristics”, “factors” or “parameters” (e.g., mixtures) may require revision.
8	The templates are not currently designed to capture data from in vivo toxicology studies.
9	Manually populating the Excel templates is time consuming and error prone.

#### Integrating data collected using the NanoPUZZLES templates and business rules into databases

Various options currently exist, or are under development, for submitting the ISA-TAB-Nano files generated using the resources presented in sections 3, 4 and (if relevant) 5 to online, searchable databases. Submission to these databases should assist nano-QSAR researchers in identifying and retrieving data for modelling.

One option, as discussed previously, would be to submit the files to a database developed using the freely available “Nanomaterial Data Management System” (“nanoDMS”) software [30,107–110] which was created within the context of the MODERN project. This database system was specifically designed to act as a searchable, online repository for ISA-TAB-Nano files and upload to the system is only allowed if the internal ISA-TAB-Nano dataset validator, also available as a standalone online tool [107], does not generate any error messages. An existing implementation of such a database was publicly available at the time of writing [110] and submission of a suitably prepared version of the “Toy Dataset” described in section 6 was successful (see Figures 3–7). However, as discussed in section 5 and section 6, this submission would currently require some modification of the datasets, i.e., some ontology identifers would need to be truncated and Investigation file “Comment […]” rows would need to be removed.

Another possible option would be to upload datasets generated using these resources into the eNanoMapper database [31,118–119]. This might be achieved via using the eNanoMapper customisable Excel spreadsheet parser to extract data from the Excel files created directly using the NanoPUZZLES templates [120]. Alternatively, it might be possible for an ISA-TAB-Nano parser (under development within eNanoMapper at the time of writing) to parse the tab-delimited text files generated using the program described in section 5. In either case the mapping of the input files onto the internal eNanoMapper data model would be performed in a transparent way, either explicitly via a JSON configuration file or implicitly by the ISA-TAB-Nano parser [31].

A brief illustration of some of the functionality of the nanoDMS database and its use for querying data generated using the NanoPUZZLES templates and business rules is presented in Figures 3–7. However, it should be noted that an in-depth discussion of the complete functionality of the nanoDMS and eNanoMapper databases is beyond the scope of the current paper. Interested readers are referred to the cited references for further details regarding the nanoDMS [30,109–110] and eNanoMapper [31,118–119] databases.

## Conclusion

There is a clear need to capture physicochemical and toxicological nanomaterial data in consistently organised electronic datasets which can be integrated into online, searchable databases to support predictive nanotoxicology. The generic ISA-TAB-Nano specification serves as a useful starting point for constructing such datasets but additional guidance regarding how to capture different kinds of (meta)data, as reported in the nanotoxicology literature, as well as exactly which (meta)data to record in these datasets is required. The publicly available resources presented in the current publication are proposed as means of (partially) addressing these requirements as well as facilitating the creation of ISA-TAB-Nano datasets. These resources are data collection templates, corresponding business rules which extend the generic ISA-TAB-Nano specification, and Python code to facilitate parsing of these datasets and integration of these datasets within other nanoinformatics resources. Nonetheless, various challenges remain with standardised collection of data from the nanotoxicology literature which these resources cannot be claimed to have definitively solved such as the need for standardised recording of stepwise sample preparation and temporal information as well as the wider need to achieve community consensus regarding minimum information standards. Extension of these resources by the nanoinformatics community, ideally working closely with the nanotoxicology community, is anticipated to enhance their value.

## Supporting Information

Please note that in addition to the following Supporting Information files, which are versions of the “Toy Dataset” referred to in section 6, the templates and Python program described in this article are publicly available as previously explained [47,114–115].

File 1 “Toy Dataset” (i.e., not real data) created using the data collection templates.

File 2 “Toy Dataset” converted using the Python program (default options).

File 3 “Toy Dataset” converted using the Python program (“-a”, “-c” options).

File 4 Additional documentation and discussion.
