# Supplementary figures and images for: An ISA-TAB-Nano based data collection framework to support data-driven modelling of nanotoxicology
Source: Beilstein J Nanotechnol. 2015 Oct 5;6:1978–99. doi: 10.3762/bjnano.6.202 (PMC4660926; doi:10.3762/bjnano.6.202)

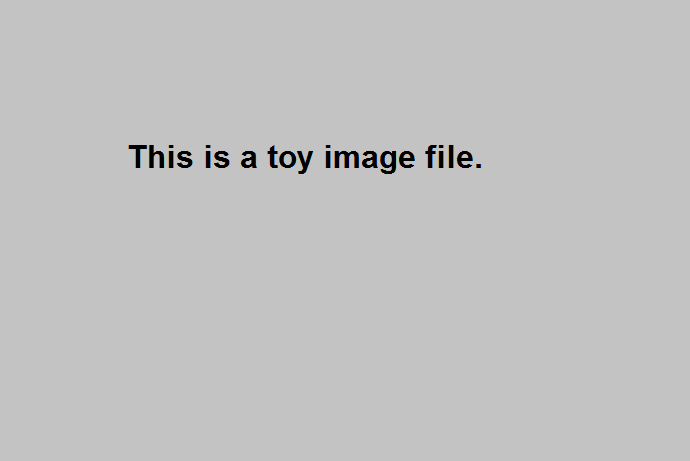

Supplement: File 1 — “Toy Dataset” (i.e., not real data) created using the data collection templates. [file Beilstein_J_Nanotechnol-06-1978-s001.zip › figure.4.A.png]
