# Supplementary material for: An ISA-TAB-Nano based data collection framework to support data-driven modelling of nanotoxicology
Source: Beilstein J Nanotechnol. 2015 Oct 5;6:1978–99. doi: 10.3762/bjnano.6.202 (PMC4660926; doi:10.3762/bjnano.6.202)
Supplement: File 4 — Additional documentation and discussion. [file Beilstein_J_Nanotechnol-06-1978-s004.pdf]

Supporting Information

for

An ISA-TAB-Nano based data collection framework to support data-driven modelling of nanotoxicology

Richard L. Marchese Robinson[1], Mark T. D. Cronin*[1], Andrea-Nicole Richarz[1], Robert Rallo[2]

Address: [1]School of Pharmacy and Biomolecular Sciences, Liverpool John Moores University, James Parsons Building, Byrom Street, Liverpool, L3 3AF, United Kingdom and [2]Departament d'Enginyeria Informatica i Matematiques, Universitat Rovira i Virgili, Av. Paisos Catalans 26, 43007 Tarragona, Catalunya, Spain

Email: Mark T. D. Cronin - M.T.Cronin@ljmu.ac.uk

*Corresponding author

Additional documentation and discussion

Contents

Supporting Information for "An ISA-TAB-Nano based data collection framework to support data driven modelling of nanotoxicology" S1
Contents S2
Supporting Information Section A. Challenges Associated with the Generic ISA-TAB-Nano Specification which were Addressed in the Current Work: In-Depth Discussion S3
Supporting Information Section B. NanoPUZZLES Business Rules: In-Depth Discussion S26
Supporting Information Section C. Some Notable Limitations of the NanoPUZZLES Templates and Business Rules Introduced in this Article: In-Depth Discussion S47
References S64

Supporting Information Section A. Challenges Associated with the Generic ISA-TAB-Nano Specification which were Addressed in the Current Work: In-Depth Discussion

Table S1 summarises the challenges with the generic ISA-TAB-Nano specification which were addressed in the current work, as per section 2 of the main text. An in-depth discussion of these challenges and the manner in which they were addressed in the current work is presented below this table. Some of the limitations of the approaches employed in the current work to address these challenges are discussed, where applicable, in the in-depth discussion of the NanoPUZZLES business rules (Supporting Information Section B) and the in-depth discussion of the "notable limitations" associated with the current work (Supporting Information Section C).

Table S1: Summary of challenges with the generic ISA-TAB-Nano specification that were addressed in the current work.

Challenge no.	Challenge	Applicable, in principle, to any format rather than being specific to ISA-TAB or ISA-TAB-Nano?	Applicable to ISA-TAB?	Applicable to ISA-TAB-Nano?
1	Standardised reporting of stepwise sample preparation needs to be established.	X	X	X
2	Ambiguity exists regarding where different kinds of information should be recorded.		X	X
3	Standardised recording of imprecisely reported experimental variables and measurements is required.	X	X	X
4	Ambiguity exists		X	X

	regarding the creation of "Comment […]" fields.			
5	Statistical terms need to be clearly defined.	X	X[a]	X[a]
6	Ambiguity exists regarding how to link to terms from ontologies.			X
7	Ambiguity exists regarding whether or not "Parameter Value" or "Factor Value" column entries must be constant or not constant respectively.		X	X
8	Linking to images reported in publications is challenging.	X	X	X
9	Standardised reporting of multiple component "characteristics", "factors", and "parameters" (e.g. mixtures) needs to be established.		X	X
10	A standardised means of linking multiple "external" files to a given Material file is required.			X
11	Greater clarity regarding the existence of "unused" factors, parameters and measurement names in the Investigation file is required.		X[a]	X
12	A standardised approach for dealing with "non-applicable" metadata is required.	X	X	X
13	The concept of an "investigation" should be more tightly defined for the purpose of collecting data from the literature.			X
14	Clearly defined minimum information criteria are required.	X	X	X

[a] It should be noted that ISA-TAB is not designed to record experimental measurements in Assay files i.e. the “Measurement Value [*statistic*(*measurement name*)]” Assay file columns and the corresponding Investigation file “Study Assay Measurement Name” field are an ISA-TAB-Nano extension [1-3]. However, regarding the issue of clearly defining statistical terms (challenge no. 5), ISA-TAB datasets may include “external” data files (i.e. “external” to the basic Investigation, Study and Assay file types) such as “data matrix” files which may include statistical terms such as “p-value” [4,5]. Standardisation of statistical terms may be achieved via using terms from the STATistics Ontology (STATO) [6]. The challenge noted here (challenge no. 5) regarding clearly defining statistical terms concerns how to appropriately create links to ontologies for these terms in ISA-TAB-Nano datasets.

(1) Standardised reporting of stepwise sample preparation needs to be established.

The preparation of nanomaterial samples (or biological samples to which nanomaterial perturbations are added) for physicochemical characterisation (or biological assays) may involve multiple processing steps being applied to a given sample as originally sourced from a vendor/manufacturer and stored for varying time periods prior to analysis [7–9]. Nanomaterial samples originally obtained from a vendor are commonly prepared as a stock suspension prior to preparing a suspension for physicochemical or biological testing [7] via steps such as diluting the stock suspension [8]. A wide variety of different processing steps may be applied to the sequentially prepared samples. For example, the stock suspension may be sonicated to varying degrees, then stored for varying time periods at varying temperatures prior to preparing a sample for physicochemical characterisation at a different temperature via vortex shaking and dilution [7,8].

However, it is arguably the case that the most recent processing history has *most* significant effect on the characteristics of the sample for which measurements are made [7,9] and the enumeration and population of fields for multiple processing steps of, potentially, the same kind (e.g. multiple sonication steps) would add significantly to the burden of data collection. Hence, within the context of the NanoPUZZLES project, it was decided to focus upon the most recent processing history. So, a single "Factor Value […]" predefined column was created in Study file templates (e.g. "Factor Value [Sonication Strength]") for each of the sample preparation variables judged to be likely to influence the resulting assay measurements[1] and the NanoPUZZLES business rules (see rule no. 2 discussed in Supporting Information Section B) specifically stipulated that they should only be used to record sample preparation variables which were applicable to the samples prepared prior to the assay protocol referenced in the corresponding Assay file. Hence, the Study file "Factor Value […]" columns would not be used to record sonication of the stock suspension as opposed to the sample prepared for testing in an assay protocol. However, any final drying step which might be applied for certain physicochemical assays (e.g. for transmission electron microscopy [8]) was considered part of the assay protocol and would be captured via setting the corresponding Study file and Assay file "Factor Value [physical state]" and "Factor Value [physical state (assay preparation)]" entries to "state of suspension" and "powdered state" respectively. (The nature of the suspension, such as the medium used to prepare the sample immediately prior to drying can have a significant effect on the images obtained from transmission electron microscopy [8].)

[1] However, as noted in section 3 of the main text and when considering challenge no. 14 below, no claim is made that the set of variables indicated to be important in the NanoPUZZLES templates is comprehensive.

Thus, the NanoPUZZLES approach focused on what are probably the most important aspects of stepwise sample preparation. Nonetheless, a more complete capturing of this information might better capture experimental variability. Possible ways of capturing this information in future work are discussed in Supporting Information Section C (limitation no. 1).

(2) Ambiguity exists regarding where different kinds of information should be recorded.

One source of potential ambiguity which is common to both ISA-TAB and ISA-TAB-Nano is the possibility to record certain kinds of experimental protocol details using Study or Assay file “Parameter Value […]” columns or using the “Study Protocol Components Name” field in the Investigation file. The ISA-TAB documentation suggests that “Study Protocol Components Name” might be used to record, amongst other protocol details, “instrument names”, yet an example is provided of a “Parameter Value [detector]” column [5]. The NanoPUZZLES Investigation file template presented in the current publication indicates (via the colour coding scheme discussed in section 3 of the main text) that the “Study Protocol Components Name” field does not need to be populated and the Assay file templates include a “Parameter Value [Instrument]” column.

One source of potential ambiguity which is specific to ISA-TAB-Nano concerns where to record different kinds of nanomaterial composition information. Notably, it is arguably unclear where certain kinds of experimentally determined or verified intrinsic chemical composition information (e.g. a dispersant aid whose presence was only revealed following experimental analysis [8]) should be recorded. (Here, the term “intrinsic” chemical composition information is used to denote information relating to the chemical composition of the originally sourced nanomaterial, as opposed to

adsorbed species in a “protein corona” [10].) As of version 1.2, the ISA-TAB-Nano specification indicates that “nominal particle characteristics (or vendor supplied) should be included in the Material File” using “Characteristics [*characteristic name*]” columns and experimentally determined characteristics should be included in an Assay file [11,12]. However, it is arguably unclear whether or not this implicitly applies to intrinsic chemical composition information which the ISA-TAB-Nano Material file was specifically designed to record via distinct field types such as the “Material Chemical Name” field used to record the chemical identities of the nanomaterial as a whole or its constituent components [1,12]. Should information which would otherwise be recorded using these distinct field types actually be recorded via Assay files if this information was experimentally determined or confirmed?

In addition, it is arguably unclear whether or not impurities should be considered “characteristics” (e.g. “Characteristics [impurities]”), in keeping with the suggestion that “purity” (of Assay file samples) might be reported via a “Characteristics [purity]” column in ISA-TAB [5], or as separate nanomaterial components which would be reported on separate rows of a Material file [12]. (In addition to this potential ambiguity, it is worth noting here that additional chemical composition information might also be recorded using “Characteristics […]” columns, such as the percentage contribution of a shell component to core/shell nanoparticles [13].)

Furthermore, the medium in which the nanomaterial was supplied (if it was not supplied as a dry powder) [8] might be treated as a different nanomaterial component [1] (i.e. a different row in a Material file) [12] or this medium might not be considered an intrinsic component of the nanomaterial (e.g. if the medium is adjusted for testing) and hence might simply be mentioned in the “Material Description” column of a Material file [12].

Within the NanoPUZZLES project, the following approach was devised to capture different kinds of intrinsic chemical composition information, based upon consideration of the issues raised above. Firstly, the following “Characteristics […]” columns were added to the Material file template to record specific kinds of chemical composition information: "Characteristics [component proportion]","Characteristics [Product impurities found {MEDDRA: http://purl.bioontology.org/ontology/MDR/10069178}]","Characteristics [Impurities proportions]"," Characteristics[purity {NPO: http://purl.bioontology.org/ontology/npo#NPO_1345}]". Secondly, a business rule (rule no. 7 discussed in Supporting Information Section B) was established which specified that any information about “impurities”, including their chemical identities, should be recorded using the "Characteristics [Product impurities found {MEDDRA: http://purl.bioontology.org/ontology/MDR/10069178}]" and "Characteristics [Impurities proportions]" columns, rather than treating the impurities as ‘normal’, distinct chemical components described via separate rows of the Material file and identified via the pre-existing “Material Chemical Name” field.

Whilst this resolves potential ambiguity regarding how to treat chemical components described as “impurities” in the publication from which data were extracted, it should be noted that the consideration of some chemical components as “impurities” may be somewhat subjective, so this approach cannot ensure that the identities of different chemical components (whether considered “impurities” or not) would be recorded consistently across all investigations. This approach would also not enable information about the nature of the linkages between impurities and the main chemical components (e.g. covalent bonding) to be described, *if* this were available, as per the linkages between the major chemical components (corresponding to separate Material file rows) which can be described via the Material file “Material

Linkage Type" field [12]. However, the current NanoPUZZLES approach to handling of impurities data does make the Material files more compact: the reporting of many impurities on separate rows of the Material file could make it harder to visually inspect the file. The limitations of the NanoPUZZLES approach to capturing intrinsic chemical composition information are returned to in Supporting Information Section C (limitation no. 4).

Thirdly, a business rule (rule no. 5 discussed in Supporting Information Section B) was developed which stipulated that any intrinsic chemical composition information associated with the a nanomaterial sample (as originally sourced) should be recorded using a Material file even if it was determined/confirmed using assay measurements reported in the publication from which the data were extracted. This approach resolves the potential ambiguity, explained above, regarding how to treat experimentally determined/confirmed intrinsic chemical composition information which would be recorded via distinct Material file fields such as "Material Chemical Name" rather than "Characteristics […]" fields. However, since certain kinds of intrinsic chemical composition information were recorded using "Characteristics […]" fields introduced within NanoPUZZLES ("Characteristics [component proportion]","Characteristics [Product impurities found {MEDDRA: http://purl.bioontology.org/ontology/MDR/10069178}]","Characteristics [Impurities proportions]"," Characteristics[purity {NPO: http://purl.bioontology.org/ontology/npo#NPO_1345}]"), this approach also meant that these fields were populated with experimentally determined values if these were available, in contrast to the standard ISA-TAB-Nano approach (as of version 1.2) that "Characteristics […]" fields should only report nominal/vendor supplied information. (All other NanoPUZZLES Material file "Characteristics […]" fields would only be populated with nominal/vendor supplied values, in keeping with the standard ISA-

TAB-Nano approach, with the Assay file templates described in section 3 of the main text designed to record corresponding experimental data.) Whilst this approach has the advantage of ensuring that all intrinsic chemical composition information was recorded together in the Material file, its main disadvantage is that nominal/vendor supplied and experimentally determined chemical composition information can only be distinguished via free text entries in “Comment […]” columns and via documenting this information in the “Material Description” field. Within the NanoPUZZLES project, free-text descriptions were recorded using “Comment […]” columns, which were added “on-the-fly”, to capture this kind of information in Material files, along with documenting this information using the “Material Description” field, but a more formalised system might be worth developing in future work. The limitations of the NanoPUZZLES approach to capturing intrinsic chemical composition information are returned to in Supporting Information Section C (limitation no. 4).

Fourthly, a business rule (rule no. 6 discussed in Supporting Information Section B) was developed specifying that any suspension medium associated with the nanomaterial sample (as originally sourced) should only be described using the Material file “Material Description” column. This avoids any potential ambiguity regarding whether or not it should be treated as another material component.

(3) . Standardised recording of imprecisely reported experimental variables and measurements is required.

In some journal articles (or scientific reports) experimental variables (e.g. “…and then probe sonicated for 30 s at 35–40W…” [8]) or assay data points (e.g. lowest observed effect level (LOEL) < 60 μg/ 10^6 cells or LOEL 30-60 μg/ 10^6 cells [14]) may be reported as ranges or limits (i.e. greater than or less than) rather than being precisely specified. A standardised means of reporting this information is required.

Within NanoPUZZLES, new business rules (rules no. 11 and 12 discussed in Supporting Information Section B) were created to address these scenarios. These stipulated that imprecisely reported experimental variables should be reported using “Factor Value [statistic(original factor name)]” columns created “on-the-fly”. For example, if sonication strength was reported as a range of values such as “35-40 W”, the predefined Study file “Factor Value [Sonication Strength]” column would be supplemented with “Factor Value [minimum(Sonication Strength)]” and “Factor Value [maximum(Sonication Strength)]” columns used to record the lower and upper limits of the range respectively. To ensure compliance with the generic ISA-TAB-Nano specification, the corresponding Investigation file “Study Factor Name” row (associated with the relevant Study file) would need to be populated with the new factor names (e.g. “minimum(Sonication Strength)” and “maximum(Sonication Strength)”) although the corresponding Investigation file “Study Factor Type” row entries should be populated as per the entry corresponding to the original factor name (e.g. “Sonication Strength”).

These business rules further stipulated that imprecisely reported measurement values should be reported using “Measurement Value [statistic(measurement name)]” columns created “on-the-fly”. For example, if a LOEL value was reported as “< 60 μg/10^6 cells” and/or “> 30 μg/10^6 cells”, the predefined “Measurement Value [mean(LOEL)]” column would be supplemented with “Measurement Value [Less Than(LOEL)]” and “Measurement Value [Greater Than(LOEL)]” columns.

(4) Ambiguity exists regarding the creation of “Comment […]” fields.

The official ISA-TAB-Nano documentation (at the time of writing) suggested [2,3,12,15] that “Comment […]” fields could only be created for Study or Assay files and the addition of “Comment [*…*]” rows to the Investigation file was not mentioned in the original ISA-TAB documentation [5]. Indeed, the MODERN project tools [16,17] for parsing ISA-TAB-Nano files do not currently support any Investigation file “Comment […]” rows. However, correspondence with the ISA-TAB-Nano developers indicated “Comment [*…*]” columns (rows) could be added to a Material, Study or Assay file (Investigation file) to record any additional information that could not be recorded using a predefined field or field type (e.g. “Factor Value [*factor name*]”). Furthermore, the ToxBank [18,19] ISA-TAB templates [20] include predefined “Comment [*…*]” rows in the Investigation file template “investigation.xml” (e.g. "Comment [Created with configuration]") and ISA-TAB Investigation file “Comment […]” rows are permitted by the ISA-Tools software [21,22]. Hence, the allowed inclusion of these additional rows or columns in all four ISA-TAB-Nano file types was explicitly stipulated in the NanoPUZZLES business rules (rule no. 13 discussed in Supporting Information Section B), along with the creation of certain predefined “Comment […]” columns or rows in the relevant template files to record certain kinds of metadata.

(5) Statistical terms need to be clearly defined.

The *statistic* terms used to describe data points obtained from assay measurements (“Measurement Value [*statistic*(*measurement name*)]”), or for experimental variables (“Factor Value [*statistic*(*original factor name*)]”) according to the NanoPUZZLES business rules (rule no. 11 discussed in Supporting Information Section B), should be clearly defined. To facilitate this, these terms should be linked to definitions from ontologies if possible e.g. the STATistics Ontology (STATO) [6]. However, whilst this

was possible in earlier versions [1], this was not possible as of version 1.2 of the generic ISA-TAB-Nano specification.

Hence, new predefined “Comment […]” rows were added to the Investigation file template developed in the current work: “Comment [Statistic name]”, “Comment [Statistic name Term Accession Number]” and “Comment [Statistic name Term Source REF]”. The NanoPUZZLES business rules stipulated that all *statistic* names should be entered in the “Comment [Statistic name]” row with the corresponding rows used to establish links to terms from ontologies where these exist. N.B. These new rows were only inserted as “Comment […]” fields to avoid inconsistencies with software designed to parse generic ISA-TAB-Nano Investigation files which do not contain “Statistic name …” rows. As discussed above (challenge 4), not all software which currently exists to parse ISA-TAB-Nano files would accept these additional rows in any case due to ambiguity regarding whether or not “Comment […]” rows can be added to the Investigation file.

(6) Ambiguity exists regarding how to link to terms from ontologies.

In Thomas et al. [1], the “Term Accession Number” fields used to link terms to corresponding ontology classes are suggested to be populated using the “identification number” of the corresponding ontology “term” (or “class”) [23] – a concept which was inherited from ISA-TAB. However, how this is carried out in practice may vary. For example, when using the ISA-Tools [21,22] program ISAcreator (version 1.7.7) to create ISA-TAB files, terms retrieved from the online ontologies resource BioPortal [24,25] result in the “Term Accession Number” being populated with the corresponding complete BioPortal ID e.g. "http://purl.obolibrary.org/obo/UO_0000032" for the Units of Measurement Ontology class with a “preferred name” of “hour” (as shown in the example ISA-TAB files

distributed with this program). However, Thomas et al. [1] indicates that the suffix of this complete ID (e.g. “UO_0000032”) may be used.

This ambiguity was addressed via stipulating, in the NanoPUZZLES business rules (rule no. 15 presented in Supporting Information Section B), that, when linking to terms from ontologies, the ontology term defined “preferred name” should be selected and the full ID entered in the corresponding “Term Accession Number” field.

(7) Ambiguity exists regarding whether or not “Parameter Value” or “Factor Value” column entries must be constant or not constant respectively

The original ISA-TAB-Nano publication [1] describes “parameters” as experimental variables which “are kept constant in an assay experiment”, while “study factors” are experimental variables which “are changed for studying their effects”. Taking into account the original ISA-TAB specification documentation [5], it would seem to be implicit that, for ISA-TAB-Nano, “Parameter Value […]” column entries should be constant when associated with a given “Protocol REF” value and “Factor Value […]” column entries should not be constant. (The former inference cannot be made for the original ISA-TAB specification: ISA-TAB files distributed with the ISAcreator software version 1.7.7 [21] include different values for the same parameter associated with a single “Protocol REF” value.) However, for the purposes of creating templates with predefined columns, it may not be possible to impose the latter restriction without inconveniently creating multiple Study/Assay files. These issues were addressed via NanoPUZZLES business rules (rules no. 16 and 17 presented in Supporting Information Section B) which explicitly specified that “Factor Value […]” column entries are allowed to be constant and only “Parameter Value […]” column entries associated with a given “Protocol REF” column entry in a Study or Assay file need to be constant.

(8) Linking to images reported in publications is challenging.

The generic ISA-TAB specification (hence, implicitly, the generic ISA-TAB-Nano specification) [5] allows one or more image files to be associated with one or more assay measurements via associating the corresponding “Sample Name” identifier with file names (if the image file is redistributed as part of the dataset) or uniform resource identifiers (URIs), meaning web-addresses in the current context, reported in the “Image File” column of the relevant Assay file. This is illustrated, for one scenario, in Table S2.

Table S2: Linking of a single measurement to multiple image files in an Assay file in accordance with the generic ISA-TAB-Nano specification. The association of different image files with the same “Sample Name” identifier is in keeping with the generic ISA-TAB specification [5], whilst the inclusion of the “Measurement Value […]” column is an ISA-TAB-Nano extension [1]. N.B. Strictly speaking, the “Image File” values are indirectly associated with the corresponding “Sample Name” identifier, as they are directly associated with “Assay Name” identifiers, according to the generic ISA-TAB specification [5]. However, it should be noted that the NanoPUZZLES implementation of ISA-TAB-Nano, in keeping with many other existing implementations [1,2,26,27], does not employ a unique “Assay Name” identifier for each assay data point, so mapping the “Image File” value directly to an “Assay Name” identifier would not allow the correspondence to a specific “Sample Name” identifier to be maintained.

Sample Name	Protocol REF	Parameter Value […]	Assay Name	Factor Value […]	Measurement Value […]	Image File
s_1	…	…	…	…	1.8	file.01.jpg
s_1	…	…	…	…	1.8	file.02.jpg
s_1	…	…	…	…	1.8	http://location-of-file.03.com

However, when recording data reported in the nanotoxicology literature, it might not be possible to create a copy of an image file corresponding to a given assay data point which can be redistributed as part of an ISA-TAB-Nano dataset (due to copyright restrictions). Nor is it necessarily the case that the relevant image will be uniquely identified via a URI. This would be the case if an image associated with a given assay data point was part of a single image file presenting multiple images, each of which correspond to assay data points for different experimental samples. These images might only be differentiated by different labels or positions. For example, Figures 1 and 2 in Murdock et al. both present transmission electron microscopy (TEM) images corresponding to different nanomaterial samples as a single file [8].

Hence, neither of the existing approaches for linking assay data to images envisaged by the generic ISA-TAB-Nano specification (referring to a redistributed file name or a URI which uniquely identifies the image) would allow for the creation of the required links between assay measurements, made for a specific prepared sample, and a specific image reported in a publication under all applicable circumstances.

In the context of the NanoPUZZLES project, the creation of a new "ImageLink" file type (see section 3 of the main text), which would be referred to in the relevant Assay file "Image File" column entry, was designed to address this issue. As stipulated in the NanoPUZZLES business rules (rule no. 18 which is explained in detail in Supporting Information Section B), this "ImageLink" file contains one row per image linked to the corresponding assay measurement (i.e. Assay file row), each of which is identified via a combination of a "Reference URI" – e.g. the URI of the composite image file containing the image of interest if this exists - and an "Image Name" that should allow the image of interest to be uniquely identified e.g. "Figure 2 (A)" if the "Reference URI" corresponded to "Figure 2".

(9) Standardised reporting of multiple component "characteristics", "factors", and "parameters" (e.g. mixtures) needs to be established.

Some experimental "factors" (e.g. "Factor Value [vehicle serum]"), "parameters" (e.g. "Parameter Value [negative control]") or "characteristics" (e.g. "Characteristics [phenotype]") might comprise multiple components. This would occur in the case of mixtures - e.g. the "vehicle serum" in an *in vitro* cell-based assay might comprise a mixture of fetal bovine serum and horse serum [8] - or when multiple, compatible values were necessary to define a certain attribute e.g. a "phenotype" defined by "large leaf", "small stem", "small inflorescence" [28]. However, ISA-TAB-Nano inherits the ISA-TAB restriction [5] on adding multiple values to a single cell in a "Factor Value […]", "Parameter Value […]" or "Characteristics […]" column [12,15,27]. Indeed, the best way to record multiple component entries for ISA-TAB Study or Assay file columns is an ongoing topic of discussion within the community [28,29]. It is important to establish a standardised approach to these scenarios to facilitate data analysis. Furthermore, if some fields are documented to report corresponding information (e.g. "Factor Value [vehicle serum]" and "Factor Value [vehicle serum heat treatment]"), corresponding multiple values should be reported consistently. One possible approach to this issue would be to repeat the "Sample Name" identifier, on different rows for Study and Assay files, as many times as there are different components in a given "Factor Value […]" column (or a given "Parameter Value […]" or "Characteristics […]" column) as per the ISA-TAB [5] approach to linking multiple images to the same "Sample Name" identifier shown in Table S2. If this approach were adopted, the relevant "Material Name" in the Material file would similarly need to be repeated on different rows for multiple component "Characteristics […]" entries. However, this approach might be rather unwieldy (especially if the ISA-TAB-Nano files were manually created as per the current work) i.e. many rows might need to be

essentially duplicated (with only the relevant “Characteristics […]”, “Parameter Value […]” or “Factor Value […]” column entries changing) in some Study, Assay or Material files.

An alternative approach, which was adopted within NanoPUZZLES, would be to establish clearly documented conventions for adding multiple values to a single cell in a “Factor Value […]”, “Parameter Value […]” or “Characteristics […]” column. Specifically, the NanoPUZZLES business rules (rule no. 3 discussed in Supporting Information Section B) stipulated that, if the entry for a “Characteristics […]”, “Factor Value […]” or “Parameter Value […]” column corresponded to multiple components, this should be recorded as a semi-colon (“;”) delimited list of the separate components.

The NanoPUZZLES business rules (rule no. 4 discussed in Supporting Information Section B) also aimed to address the scenario of how to populate corresponding fields when the field to which they refer contains multiple values: the use of consistently ordered semi-colon (“;”) delimited lists was advocated. For example, the *in vitro* (cell-based) Study file in the “Toy Dataset” discussed in section 6 of the main text and available from the Supporting Information (“s_TOY.article_InVitro.CB.xls”) contains a “Factor Value [vehicle serum]” entry “fetal bovine serum; horse serum”: the corresponding “Factor Value [vehicle serum heat treatment]” entry was “TRUE;FALSE”.

However, it should be noted that the scenario of corresponding fields which refer to fields with multiple values would still present problems with data analysis. For example, the existing NanoPUZZLES approach results in some column entries being populated with mutually exclusive entries which would not have a clear semantic interpretation if these entries were parsed in isolation e.g. the “TRUE;FALSE”

example presented above. The challenges associated with implementing this business rule are returned to in Supporting Information Section C (limitation no. 7).

(10) A standardised means of linking multiple "external" files to a given Material file is required.

The generic ISA-TAB-Nano specification provides a Material file field ("Material Data File") which can be used to link to "external" data files. However, this field is documented [12] as not being permitted to specify the name of more than one file per cell. In practice, this might be valuable. For example, the nanomaterial might be associated with multiple (partial) structural representations which might be used to calculate (different kinds of) descriptors for nano-QSAR development amongst other possibilities: Simplified Molecular Line Entry System (SMILES) [30–32]; "SMILES-like" string representations [33,34]; crystallographic information files (CIF) [35] for storing unit cell parameters [36]; directed acyclic graph string representations [37] etc.

The NanoPUZZLES business rules proposed (rule no. 19 presented in Supporting Information Section B) that all files (e.g. SMILES or CIF files), corresponding to different (partial) representations of a given nanomaterial's structure, or the structure of a specific component, should be included in a single flat, compressed ZIP archive and the name of this archive should be referred to in the first applicable row of the "Material Data File" column of the relevant Material file. Each file in this ZIP archive should be clearly described using the corresponding "Material Data File Description" entry and, where possible, standard file extensions should be used e.g. ".smi" for SMILES files.

(11) Greater clarity regarding the existence of "unused" factors, parameters and measurement names in the Investigation file is required.

All "factors", or "parameters" associated with a given protocol, for a given study must be reported in the relevant Investigation file "Study Factor Name" or "Study Protocol Parameters Name" rows i.e. "Factor Value [*factor name*]" or "Parameter Value [*parameter name*]" Study or Assay file columns are only allowed if the "*factor name*" or "*parameter name*" is defined in the corresponding Investigation file [2,3,15]. Likewise, all "*measurement name*" values corresponding to Assay file "Measurement Value[*statistic*(*measurement name*)]" columns should be reported in the corresponding Investigation file "Study Assay Measurement Name" field. However, if Study or Assay file templates with predefined "factors", "parameters" and "measurement names" are used, as per the NanoPUZZLES templates described in section 3 of the main text, corresponding values may not be available for a given dataset. Hence, it might be convenient to delete the corresponding "Factor Value [*factor name*]", "Parameter Value [*parameter name*]" or "Measurement Value[*statistic*(*measurement name*)]" columns without having to update the Investigation file if those templates are manually populated as per the NanoPUZZLES templates described in section 3 of the main text – rather than using software which would automatically keep the different files consistent (see limitation no. 9 in Supporting Information Section C).

The NanoPUZZLES business rules (rule no. 20 discussed in Supporting Information Section B) explicitly allowed for this. A disadvantage of allowing for this is that an Investigation file with orphaned "factors", "parameters" or "measurement names" would give a misleading indication of the (meta)data content of the dataset – although this problem also exists with the generic ISA-TAB-Nano specification if empty columns are not deleted.

(12) A standardised approach for dealing with “non-applicable” metadata is required.

Some Study file metadata fields (e.g. certain “Characteristics […]” or “Factor Value […]” columns) may not be applicable to all samples being described in a given Study file. For example, the Study file template designed for physicochemical studies in the current work (see section 3 of the main text) includes the predefined column "Factor Value [probe]" which denotes the “probe” species (e.g. chlorobenzene) for which adsorption to the nanomaterial will be measured [38] in an adsorption assay. However, this is only applicable if the prepared sample, denoted by the corresponding “Sample Name” identifier, is being prepared prior to the application of an adsorption assay protocol. Under these circumstances, an empty column entry could convey that the corresponding information was absent, which would mislead the end user of the dataset.

The NanoPUZZLES business rules (rule no. 21 discussed in Supporting Information Section B) proposed that non-applicable columns should be populated with “N/A”, where this conveys information i.e. not in cases, such as physicochemical Study file “Factor Value [medium]” entries corresponding to the “Factor Value [physical state]” value “powdered state”, where entering “N/A” would arguably be redundant and this would add to the burden of curation. In these latter cases, it might be worthwhile to develop software to auto-assign “N/A” values in future work to facilitate automated assessment of (meta)data completeness e.g. code might be written which would automatically set a “Factor Value [medium]” entry to “N/A” if the corresponding “Factor Value [physical state]” entry read “powdered state”.

(13) The concept of an "investigation" should be more tightly defined for the purpose of collecting data from the literature.

The generic ISA-TAB-Nano specification does not tightly define what an "investigation" is [1,5,39]. The original ISA-TAB specification document suggests that the "Investigation" section of an Investigation file is "a flexible mechanism for grouping two or more Study files where required" [5]. Arguably, a variety of different biological and/or physicochemical studies on any arbitrary combination of nanomaterials might be grouped as a single "investigation". When collecting data from the published literature, for example, one might consider (subsets of) data reported in an arbitrary combination of one or more publications as a single "investigation". More specific guidance would be useful when collecting data from the literature for the following reasons: (1) associating the Material files with the minimum number of literature citations possible would make the provenance of their data clearer; (2) grouping of publications which explicitly refer to studies on identical nanomaterials has implications as to which nanomaterials may be considered identical. For example, if information about the same nanomaterials (i.e. the same originally sourced samples as declared by the authors) was reported in different publications (e.g. as per Puzyn et al. [36] and Hu et al. [40]), the ability to identify the nanomaterials as the same might be adversely affected if different investigations were created for each publication (i.e. different Material files were created for the same nanomaterial for each nanomaterial-publication combination) since the identification of two nanomaterials as "the same" is arguably an unresolved challenge. Hence, when collecting data from the literature, it is arguably the case that a single "investigation" must be based upon at least the relevant publications reporting different information for the same nanomaterials (as identified by the authors of those publications).

Within NanoPUZZLES, this issue was addressed via stipulating (rule no. 1 presented in Supporting Information Section B) that a new “investigation” should be created for each reference (e.g. journal article), unless that reference specifically states that additional information regarding experiments on the same original nanomaterial samples was reported in another reference. In the latter case, the “investigation” was proposed to correspond to both of these references.

(14) Clearly defined minimum information criteria are required.

The issue of which (meta)data “must” be reported in a nanomaterial data resource encompasses the necessary physicochemical characterisation parameters which should be available in order to reduce uncertainty in the interpretation of results, discern whether (essentially) the same nanomaterials have been tested in different studies and/or allow for structure-activity relationships to be developed [41,42].

It also encompasses the question of which experimental variables (e.g. cell line [43] or temperature) have the most impact upon the variability in the results. The values of these variables are arguably critical in order to see whether data heterogeneity [44] is sufficiently small that data from multiple sources (as opposed to data from a single source [45]) could be combined into a single dataset for building a (nano-)QSAR.

(N.B. Whilst a single “source” of data might simply refer to data reported in a single publication [45], a single “source” of data in the current context should be understood to refer to any individual collection of data, from a single publication or a single electronic dataset, generated according to the same experimental protocols carried out under the same conditions in the same laboratory.)

The question of which biological endpoints should be recorded also arises [46,47].

This general issue may also be considered to encompass additional metadata

requirements that may be required for nanotoxicology data to be considered of high quality [45].

The question of which physiochemical characteristics, experimental variables or biological endpoints should be recorded within a dataset lies beyond the scope of the generic ISA-TAB-Nano specification [1,5,39]. Whilst the generic specification does provide pre-defined Investigation file fields [3] to support some of the additional metadata requirements (such as provenance) which might be necessary for assessing the “reliability” of the data [45], it does not contain predefined fields for other requirements such as whether or not the data were generated according to Good Laboratory Practice (GLP) [45,48]. Furthermore, even given a list of minimum information criteria, determining exactly how to create the requisite ISA-TAB-Nano fields to ensure this criteria are met when recording data can be a challenge - especially if definitions from ontologies are sought. Hence, the creation of mandatory fields (based on the ISA-TAB-Nano specification e.g. specific predefined “Factor Value […]” columns for some experimental variables), specifying which characterisation parameters and experimental variables should be reported is critically important. The templates created in the work reported in this publication (see section 3 of the main text) were intended to address these requirements.

However, no claim is made to have definitively addressed this issue. Indeed, the issue of which are the most important (meta)data for nanotoxicology data sets is one which remains a subject of considerable debate within the nanoinformatics and, indeed, the nanoscience community with no definitive consensus [41,49].

Supporting Information Section B. NanoPUZZLES Business Rules: In-Depth Discussion

Table S3 summarises the business rules developed within NanoPUZZLES, as per section 4 of the main text, whilst the following discussion elaborates upon these rules: both clarifying their meaning and also discussing their key strengths and weaknesses where possible alternatives merit consideration in future work.

Table S3: Summary of the NanoPUZZLES business rules.

Business rule no.	Short description
1	A new "investigation" (corresponding to a new dataset comprising a single Investigation file, a set of Study, Assay and Material files and any "external" files if applicable) should be created for each reference (e.g. journal article), unless that reference specifically states that additional information regarding experiments on the same original nanomaterial samples was reported in another reference.
2	The "Factor Value […]" columns in the Study file refer to those values which are applicable to the sample prepared immediately prior to application of an assay protocol.
3	If the entry for a "Characteristics […]", "Factor Value […]" or "Parameter Value […]" column corresponds to multiple components (e.g. mixtures), record this as a semi-colon (";") delimited list of the separate components.
4	If the entry for a "Characteristics […]", "Factor Value […]" or "Parameter Value […]" column corresponds to multiple components, record the entries in corresponding columns as a semi-colon (";") delimited list with the entries in the corresponding order.
5	Any intrinsic chemical composition information associated with a nanomaterial sample (as originally sourced) should be recorded using a Material file even if it is determined/confirmed using assay measurements reported in the publication from which the data were extracted.
6	Any suspension medium associated with the nanomaterial sample (as originally sourced) should only be described using a Material file "Material Description" column.
7	Any impurities should be described using entries in the relevant Material file "Characteristics [….]" columns.
8	Any original nanomaterial components, which are neither a suspension medium nor described as "impurities" in the reference from which the data are extracted, should be described using separate rows of the Material file as per the generic ISA-TAB-Nano specification.
9	All "Sample Name" values for "true samples" should have the following form: "s_[Study Identifier]_[x]" e.g. "s_[Study Identifier]_1".[a]

10	Assay file "Measurement Value […]" column entries which correspond to concentration-response curve statistics, or similarly derived measures, should be associated with a "derived sample" identifier rather than a "true sample" identifier.
11	Imprecisely reported experimental variables should be reported using "Factor Value [statistic(original factor name)]" columns created "on-the-fly".
12	Imprecisely reported measurement values should be reported using "Measurement Value [statistic(measurement name)]" columns created "on-the-fly".
13	"Comment […]" columns (rows) can be added without restriction to a Study, Assay, Material (Investigation) file as long as they are appropriately positioned and as long as each new "Comment […]" column (row) has a unique name for a given file.
14	All "statistic" names must be entered in the corresponding Investigation file template "Comment [Statistic name]" row.
15	When linking to terms from ontologies, the "preferred name" should be selected and the full ID entered in the corresponding "Term Accession Number" field.
16	"Factor Value […]" column entries are allowed to be constant.
17	Only "Parameter Value […]" column entries associated with a given "Protocol REF" column entry in a Study or Assay file need to be constant.
18	Images should be linked to assay measurements using a new "ImageLink" file type, if the generic ISA-TAB-Nano approach cannot be applied.
19	Any nanomaterial structure representation files, which are not associated with specific Assay file "Measurement Value […]" entries, should be linked to the corresponding Material file using ZIP archives specified in the appropriate "Material Data File" column entry.
20	Empty "Factor Value […]", "Parameter Value […]" or "Measurement Value […]" columns in Study or Assay files can be deleted without having to update the corresponding Investigation file "Study Protocol Parameters Name", "Study Factor Name", or "Study Assay Measurement Name" fields.
21	Non-applicable columns should be populated with "N/A" where this conveys information.
22	"Measurement Value [statistic(measurement name)]" columns in the templates which use a label of the form "[TO DO:…]" for the statistic or measurement name must either be updated, based on the kind of statistic and/or measurement name indicated by the label(s), or deleted.

[a] Here, the "[Study Identifier]" [3] is unique to the corresponding Study file and "[x]" denotes a numeric value which is specific to a given "true sample", meaning a prepared sample corresponding to a specific set of experimental conditions, in contrast to the "derived sample" concept introduced in NanoPUZZLES business rule no. 10.

(1) A new "investigation" (corresponding to a new dataset comprising a single Investigation file, a set of Study, Assay and Material files and any "external" files if applicable) should be created for each reference (e.g. journal article), unless that reference specifically states that additional information regarding experiments on the same original nanomaterial samples was reported in another reference.

In the latter case, the "investigation" should correspond to both of these references. N.B. As explained in section 1 of the main text, an "external" file denotes any other file included in the dataset which is not an Investigation, Study, Assay or Material file.

(2) The "Factor Value […]" columns in the Study file refer to those values which are applicable to the sample prepared immediately prior to application of an assay protocol.

Hence, values which are only applicable to the stock suspension of a nanomaterial, prepared prior to deriving a suspension for testing, should not be recorded using these columns. Values which are associated with an assay protocol should not be recorded using these columns, but should be recorded using the applicable Assay file columns. For example, the values appropriate to the nanomaterial suspensions prepared prior to drying for transmission electron microscopy measurements [8] should be recorded using Study file "Factor Value […]" columns (e.g. the "Factor Value [physical state]" column entries would read "state of suspension") whilst the drying step would be captured via the relevant Assay file "Factor Value [physical state (assay preparation)]" column entry: "powdered state".

(3) If the entry for a "Characteristics […]", "Factor Value […]" or "Parameter Value […]" column corresponds to multiple components (e.g. mixtures), record this as a semi-colon (";") delimited list of the separate components.

For example, if the “vehicle serum” in an *in vitro* cell-based assay comprised a mixture of fetal bovine serum and horse serum [8], the relevant entry in the “Factor Value [vehicle serum]” column would be populated with “fetal bovine serum; horse serum”.

(4) If the entry for a “Characteristics […]”, “Factor Value […]” or “Parameter Value […]” column corresponds to multiple components, record the entries in corresponding columns as a semi-colon (“;”) delimited list with the entries in the corresponding order.

For example, the *in vitro* cell-based Study file in the “Toy Dataset” discussed in section 6 of the main text and available from the Supporting Information (“s_TOY.article_InVitro.CB.xls”) contains a “Factor Value [vehicle serum]” entry “fetal bovine serum; horse serum”: the corresponding “Factor Value [vehicle serum heat treatment]” [8] entry was “TRUE;FALSE”.

However, this business rule has certain disadvantages e.g. it would lead to column entries (such as “TRUE;FALSE” in the example presented here) which have no clear semantic meaning if parsed in isolation. The challenges associated with this business rule are returned to under Supporting Information Section C (limitation no. 7). Nonetheless, no clear alternative rule which would address this issue currently exists.

(5) Any *intrinsic* chemical composition information associated with a nanomaterial sample (*as originally sourced*) should be recorded using a Material file even if it is determined/confirmed using assay measurements reported in the publication from which the data were extracted.

This includes the chemical identity and relative proportions of any chemical components (e.g. core, coat, dispersant aids [8], impurities etc.), along with information about how different components are linked, associated with the

nanomaterial samples originally sourced for a set of experiments. Hence, in contrast to the generic ISA-TAB-Nano specification [11,12], this would also apply to the new Material file “Characteristics […]” columns introduced to report certain kinds of chemical composition information: "Characteristics [component proportion]","Characteristics [Product impurities found {MEDDRA:http://purl.bioontology.org/ontology/MDR/10069178}]","Characteristics [Impurities proportions]","Characteristics[purity {NPO: http://purl.bioontology.org/ontology/npo#NPO_1345}]". However, in keeping with the generic ISA-TAB-Nano specification (as of version 1.2), all other “Characteristics […]” columns would only be populated with nominal/vendor supplied information.

N.B. Whilst this business rule has the advantage of ensuring that all intrinsic chemical composition information is recorded within the Material file, its disadvantage is that experimentally determined/verified composition information could only be distinguished from nominal/vendor supplied information using free text descriptions presented in “Comment […]” columns and as part of the “Material Description” field entries. However, it should be remembered that this business rule only applies to the specific case of experimentally determined, intrinsic chemical composition information. This does not include adsorption (e.g. protein corona) data [10,38], which should be recorded using the Assay file template “a_InvID_PC_adsorption_Method.xls”. Nor does this include any other experimentally determined physicochemical properties, which would be recorded using the appropriate Assay file template (see Table 2 in the main text). Hence, for all other experimentally determined physicochemical data other than intrinsic chemical composition information, the experimental conditions (e.g. the medium) under which the characteristic (e.g. size) was measured, and the experimental technique (e.g. dynamic light scattering), should be fully documented in a standardised manner using

the appropriate fields in the Investigation file, physicochemical Study file and Assay file as explained in section 3 of the main text.

(6) Any suspension medium associated with the nanomaterial sample (*as originally sourced*) should only be described using a Material file "Material Description" column.

For example, when creating Material files for the nanoparticles received as suspensions in water by Murdock et al. [8], water was not treated as a chemical component but was only referred to in the "Material Description" column.

(7) Any impurities should be described using entries in the relevant Material file "Characteristics [….]" columns.

These relevant columns are the "Characteristics [Product impurities found {MEDDRA:http://purl.bioontology.org/ontology/MDR/10069178}]" and "Characteristics [Impurities proportions]" columns. These entries should be associated with the initial Material file rows, which refer to the nanomaterial sample as a whole [12], rather than rows corresponding to constituent components *unless* the publication from which the data are extracted specifically indicates the impurities are associated with a given component.

For example, if the Material file template is used to describe a metal oxide nanomaterial sample with iron (0.1%) and silver (0.2%) impurities on the surface, the column "Characteristics [Product impurities found {MEDDRA:http://purl.bioontology.org/ontology/MDR/10069178}]" would contain the entry "iron; silver" and the corresponding "Characteristics [Impurities proportions]" and associated "Unit" column entries would read "0.1;0.2" and "percent; percent" respectively. This example is presented in one of the Material files ("m_TiO2_TOY.article.xls") contained within the "Toy Dataset" discussed in section 6 of the main text and available from the Supporting Information (see Table S4).

Table S4: Part of one of the Material files ("m_TiO2_TOY.article.xls") available from the Supporting Information, illustrating how surface impurities data would be recorded according to the current NanoPUZZLES business rules. N.B. (1) Only the rows corresponding to the core and shell components are shown i.e. not the initial rows corresponding to this hypothetical nanomaterial as a whole, which report a purity value of 99 percent via the "Characteristics [purity {NPO: http://purl.bioontology.org/ontology/npo#NPO_1345}]" and associated "Unit" fields. (2) Column names have been truncated due to space constraints.

Material Source Name	Material Name	Material Type	Characteristics [Product impurities found ….]	Characteristics [Impurities proportions]	Unit
TiO2_TOY.article	TiO2_core	core; metal oxide			
TiO2_TOY.article	silica_shell	shell	iron;silver	0.1; 0.2	percent; percent

N.B. Since the description of some chemical components as "impurities" may be somewhat subjective, this approach cannot ensure that the identities of different chemical components (whether they were consideredd impurities or not) would be recorded consistently across all investigations. These and other limitations of the existing NanoPUZZLES business rules for handling chemical composition information are discussed in Supporting Information Section C (limitation no. 4).

(8) Any original nanomaterial components, which are neither a suspension medium nor described as “impurities” in the reference from which the data are extracted, should be described using separate rows of the Material file as per the generic ISA-TAB-Nano specification.

(9) All “Sample Name” values for “true samples” should have the following form: “s_[Study Identifier]_[x]” e.g. “s_[Study Identifier]_1”.

Here, the “[Study Identifier]” [3] is unique to the corresponding Study file and “[x]” denotes a numeric value which is specific to a given “true sample”, meaning a prepared sample corresponding to a specific set of experimental conditions (e.g. a specific “Factor Value [screening concentration]” value) in contrast to the “derived sample” concept introduced in NanoPUZZLES business rule no. 10. The rationale for this specific naming convention is also explained when discussing NanoPUZZLES business rule no. 10. These “Sample Name” identifiers should not be confused with the similarly named Study files which, according to the NanoPUZZLES naming conventions discussed in section 3 (“General Overview of Templates”) of the main text, would be named “s_[Study Identifier].<file extension>” e.g. “s_TOY.article_PC.txt” in the tab-delimited text versions of the “Toy Dataset” described in section 6 of the main text and available from the Supporting Information. It should be noted that, whilst these “Sample Name” identifiers are also conveniently created in Excel, a potential disadvantage is that they are not sufficiently descriptive: the relevant experimental details (e.g. the nanomaterial being tested) corresponding to a given Assay file measurement would need to be retrieved from the Study file via the “Sample Name” identifier. However, this disadvantage only manifests itself if examining the datasets via software (such as Excel) which can only visualise the (meta)data in individual files: software, such as future extensions of the ISA-Tools [21] to parse ISA-TAB-Nano files, which could automatically link the (meta)data and

measurement values in Study and Assay files via the corresponding “Sample Name” identifiers would not require human readable identifiers.

(10) Assay file “Measurement Value […]” column entries which correspond to concentration-response curve statistics, or similarly derived measures, should be associated with a “derived sample” identifier rather than a “true sample” identifier.

N.B. An overview of different scenarios under which this rule was applied within NanoPUZZLES is followed by (1) an explanation of how this rule would be applied under these relevant scenarios and (2) a discussion of a possible alternative which is more in keeping with the generic ISA-TAB specification and should be considered in future work.

Scenarios for which this rule should be applied

When collecting data from literature references, there are various scenarios in which reported data points are derived from measurements made for multiple samples prepared according to different values for certain experimental variables. These “derived data points” may be reported instead of or in addition to the “underlying measurements” for the different samples. For example, a LOEL [50] or LC_{50} [51] might be derived from a corresponding dose-response or concentration-response curve or, to give another example, an Ames test study call (i.e. “positive”, “negative” or “equivocal”) might be derived from measurements made at multiple concentrations, in multiple strains of different bacteria with or without “S9 mix” being included in the exposure medium [52–55].

NanoPUZZLES business rule

The “underlying measurements” (e.g. “Measurement Value [mean(percent cytotoxicity)]”) and sample specific experimental conditions (e.g. “Factor Value [screening concentration]” column entries) should be associated with “true samples”

e.g. with “Sample Name” values “s_[Study Identifier]_1”, “s_[Study Identifier]_2” and “s_[Study Identifier]_3” if three concentrations were tested to derive an LC_{50}.

The corresponding “derived data point” (e.g. “Measurement Value [mean(LC50)]”) should be associated with a “derived sample” with a “Sample Name” value named after the corresponding “true sample” names e.g. “s_[Study Identifier]_derived: 1,2,3” if the corresponding “true sample” names were “s_[Study Identifier]_1”, “s_[Study Identifier]_2” and “s_[Study Identifier]_3”.

Since the NanoPUZZLES Study file templates developed to date only contain a single “Sample Name” column, these “derived sample” identifiers would need to be reported in this column along with the corresponding “true sample” identifiers. The entries, associated with the “derived sample”, in the “Characteristics […]” and “Factor Value […]” columns corresponding to the varied experimental variables for the relevant “true samples” should be left blank. If the “derived samples” correspond to multiple values for a variable recorded using a “Source Name” associated “Characteristics […]” column (e.g. “strain”, as would be the case if test results from multiple strains of S. Typhimurium were used to derive an overall Ames test study call [52–54]), not only should the relevant “Characteristics […]” column be left blank, but a new “Source Name” would be required (“<source name 1>_and_<source name 2>”), since the “Source Name” associated “Characteristics […]” columns are used to denote intrinsic properties of the original specimen used to prepare the sample tested in some assay.

The application of this business rule is summarised in Table S 5 and Table S 6.

Table S5: Application of the existing NanoPUZZLES business rule to handling derived data such as dose response parameters or genotoxicity study calls derived from tests against multiple strains of bacteria: creation of “derived sample” identifiers in the Study file. The table is adapted from the *in vitro* cell-based Study file (“s_TOY.article_InVitro.CB.xls”) available in the Supporting Information. Due to space constraints, the “Protocol REF” columns and most columns related to non-varied experimental conditions have been removed, along with the “Factor Value [vehicle]” entries which were varied for the hypothetical samples prepared for an Ames test, and names shortened. The “true sample” and “derived sample” identifiers are linked to their corresponding “Measurement Value […]” entries in the applicable Assay files: “a_TOY.article_cytotoxicity.cell-viability_MTT.xls” (“s_...2”, “s_...3”, “s_...4”, “s_..._derived:2,3,4”), “a_TOY.article_genotoxicity_Ames.xls” (“s_...6”, “s_...7”, “s_...8”, ”s_..9”, “s_..._derived:6,7,8,9”).

Source Name	Characteristics [strain …]	Sample Name	Factor Value [nanomaterial]	Factor Value [screening concentration]
cells_1		s_..._2	TiO2_...	0.1
cells_1		s_..._3	TiO2_...	0.2
cells_1		s_..._4	TiO2_...	0.3
cells_1		s_... _derived:2,3,4	TiO2_...	
S..._TA97a	TA97a	s_..._6	TiO2_...	
S..._TA98	TA98	s_..._7	TiO2_...	
S..._TA97a	TA97a	s_..._8	TiO2_...	
S..._TA98	TA98	s_..._9	TiO2_...	
S..._TA97a _and_ S..._TA98		s_... _derived:6,7,8,9	TiO2_...	

Table S6: Linking of “true sample” (“s_..._2”, “s_..._3”, “s_..._4”) and “derived sample” identifiers to the “underlying measurements” (“percent cytotoxicity”) and “derived data points” (“LC50”) respectively, in keeping with the current NanoPUZZLES business rule, via an Assay file. The table is adapted from “a_TOY.article_cytotoxicity.cell-viability_MTT.xls” available from the Supporting Information. Various columns have been removed and names shortened due to space constraints.

Sample Name	Protocol REF	Assay Name	Measurement Value [mean(percent cytotoxicity)]	Measurement Value [mean(LC50)]
s_..._2	cell viability assay	cell viability assay	10	
s_..._3	cell viability assay	cell viability assay	40	
s_..._4	cell viability assay	cell viability assay	70	
s_...derived: 2,3,4	cell viability assay	cell viability assay		0.25

An alternative which should be considered in future work

Whilst the preceding discussion documents the business rule applied within NanoPUZZLES for handling scenarios in which data points are derived from data obtained under different experimental conditions (e.g. concentration-response curve parameters such as LC_{50} values), an alternative approach, which employs the "pooling" approach documented in the ISA-TAB specification [5], would be worth considering in future work as it offers certain advantages over the existing NanoPUZZLES business rule and is more in keeping with the generic ISA-TAB specification. This alternative would entail adapting the ToxBank approach [19,56] to capturing dose response data via ISA-TAB – bearing in mind that the ToxBank approach does not take account of the "Measurement Value […]" columns added in ISA-TAB-Nano and also relies on data point specific "Assay Name" identifiers, in contrast to the work carried out within NanoPUZZLES and many other implementations of ISA-TAB-Nano [1,2,26,27] which employed a single "Assay Name" identifier for a given Assay file. Hence, the exact manner in which the ToxBank approach to handling dose response data might be adapted, as an alternative to the existing NanoPUZZLES business rule based on "derived sample" identifiers, would require further discussions with the ISA-TAB-Nano developers. However, a possible means via which this alternative could be implemented is illustrated, for the case of LC_{50} "derived data points", in Table S 7: all NanoPUZZLES Assay file templates which currently include "Measurement Value […]" columns corresponding to "derived data points" would need to be updated to include the new "Protocol REF" and "Data Transformation Name" columns.

As well as offering various advantages, the disadvantages of this alternative would be as follows: (1) the existing NanoPUZZLES ISA-TAB-Nano templates would need updating with additional columns and data point specific "Assay Name" identifiers

would need to be assigned, in contrast to the work in contrast to the work carried out within NanoPUZZLES and many other implementations of ISA-TAB-Nano [1,2,26,27] which employed a single “Assay Name” identifier for a given Assay file; (2) the structure of the files would be further complicated by the fact that a single row would no longer correspond to a single identifier linking a biological and/or nanomaterial sample combination, evaluated under certain experimental conditions, to the outcome of an assay; (3) the “derived data points” (e.g. “Measurement Value [mean(LC50)]” entries) would need to be repeated in the Assay file as many times as there were corresponding “true samples”.

However, this alternative would also have certain advantages. Firstly, in contrast to the existing NanoPUZZLES approach of adding a new “derived sample” identifier to a single “Sample Name” column, this alternative would avoid duplication of the non-varied experimental conditions and avoid blank entries in the “Characteristics […]” and “Factor Value […]” columns corresponding to the varied experimental conditions in the Study file: these blank entries might imply missing metadata. Secondly, this alternative would be ensure that the “Sample Name” identifiers corresponding to the samples tested under the varied conditions (e.g. different “Factor Value [screening concentration]” values) and the identifiers linked to the “derived data points” (e.g. ”Measurement Value [mean(LC50)]” values) were linked via an established ISA-TAB approach for associating different file “nodes” [5], rather than relying on non-standard naming conventions introduced within the NanoPUZZLES project.

Table S7: A *possible* alternative means of capturing “derived data points” (e.g. LC_{50} estimates) within an ISA-TAB-Nano Assay file which, in contrast to the existing NanoPUZZLES business rule, does not rely on “derived sample” identifiers. The “Sample Name” identifiers correspond to the different tested concentrations. The “Measurement Value [mean(LC50)]” values could be associated with the corresponding “Data Transformation Name” identifier, the “Measurement Value [mean(percent cytotoxicity)]” values with the corresponding “Assay Name” identifier, which could in turn be associated with the corresponding “Sample Name” identifier. The “Assay Name” and “Data Transformation Name” identifiers should be unique across the entire dataset. N.B. Due to space constraints, some Assay file columns are not shown and names have been truncated.

Sample Name	Protocol REF	Assay Name	Measurement Value [mean(percent cytotoxicity)]	Protocol REF	Data Transformation Name	Measurement Value [mean(LC50)]
s_..._2	cell viability assay	A1	10	calculation	D1	0.25
s_..._3	cell viability assay	A2	40	calculation	D1	0.25
s_..._4	cell viability assay	A3	70	calculation	D1	0.25

(11) Imprecisely reported experimental variables should be reported using "Factor Value [statistic(original factor name)]" columns created "on-the-fly".

For example, if sonication strength was reported as a range of values such as "35-40 W" [8], the predefined Study file "Factor Value [Sonication Strength]" column would be supplemented with "Factor Value [minimum(Sonication Strength)]" and "Factor Value [maximum(Sonication Strength)]" columns used to record the lower and upper limits of the range respectively. To ensure compliance with the generic ISA-TAB-Nano specification, the corresponding Investigation file "Study Factor Name" row (associated with the relevant Study file) would need to be populated with the new factor names (e.g. "minimum(Sonication Strength)" and "maximum(Sonication Strength)") although the corresponding Investigation file "Study Factor Type" row entries should be populated as per the entry corresponding to the original factor name (e.g. "Sonication Strength") [3].

(12) Imprecisely reported measurement values should be reported using "Measurement Value [statistic(measurement name)]" columns created "on-the-fly".

For example, if a LOEL value was reported as "< 60 μg/10^6 cells" and/or "> 30 μg/10^6 cells" [14], the predefined "Measurement Value [mean(LOEL)]" column would be supplemented with "Measurement Value [Less Than(LOEL)]" and "Measurement Value [Greater Than(LOEL)]" columns.

(13) "Comment […]" columns (rows) can be added without restriction to a Study, Assay, Material (Investigation) file as long as they are appropriately positioned and as long as each new "Comment […]" column (row) has a unique name for a given file.

The ISA-TAB specification [5] indicates that these fields should be associated with specific Study or Assay file “nodes” (e.g. “Sample Name”). Indeed, specific ordering conventions for these columns are enforced by the tools developed within the MODERN project [16]. As a pragmatic means of facilitating integration with those tools, “Comment […]” columns in ISA-TAB-Nano Study and Assay files (created within NanoPUZZLES) are required to be positioned after the “Sample Name” column and before any other columns. Investigation file “Comment […]” rows should not come between associated rows (e.g. “Investigation Publication Status” and “Investigation Publication Status Term Accession Number”). However, it should be noted (see section 5 of the main text) that the MODERN project tools [16] do not currently support any Investigation file “Comment […]” rows.

(14) All “statistic” names must be entered in the corresponding Investigation file template “Comment [Statistic name]” row.

These “statistic” names would be found in “Measurement Value [*statistic(measurement name)*]” and, possibly, “Factor Value [*statistic(factor name)*]” columns (see NanoPUZZLES business rule no. 11). These names should be linked to ontologies, where possible, using the corresponding “Comment [Statistic name Term Accession Number]” and “Comment [Statistic name Term Source REF]” fields.

(15) When linking to terms from ontologies, the “preferred name” should be selected and the full ID entered in the corresponding “Term Accession Number” field.

For example, consider retrieving the term “titanium oxide nanoparticle” [57] for a Material file “Material Chemical Name” column entry from the NanoParticle Ontology (NPO) [23] via BioPortal [24,25]. The “Preferred Name” value (i.e. “titanium oxide nanoparticle”), rather than any of the “Synonyms” values (i.e. “TiO2 nanoparticle” in the current case) should be entered in the “Material Chemical Name” column, the full

“ID” value (“http://purl.bioontology.org/ontology/npo#NPO_1486”) should be entered in the adjacent “Term Accession Number” column and the abbreviated ontology name (“NPO”) defined in the Investigation file would be entered in the corresponding “Term Source REF” column.

(16) “Factor Value […]” column entries are allowed to be constant.

(17) Only “Parameter Value […]” column entries associated with a given “Protocol REF” column entry in a Study or Assay file need to be constant.

(18) Images should be linked to assay measurements using a new “ImageLink” file type, if the generic ISA-TAB-Nano approach cannot be applied.

The relevant Assay file “Image File” column entry should either report (1) the name of a single image file redistributed as part of the ISA-TAB-Nano dataset, (2) a uniform resource identifier (URI) which links to a single image file, or (3) the name of an “ImageLink” file which is redistributed as part of the current ISA-TAB-Nano dataset. Approaches (1) and (2) are consistent with the generic ISA-TAB-Nano specification [2] (see Table S2). Approach (3), which is described below, should only be applied if approaches (1) or (2) cannot be applied.

This “ImageLink” file should then contain rows corresponding to each of the associated images. The columns in the “ImageLink” file should be populated as follows (Table S 8). “Image ID” should report unique, consecutive IDs e.g. “1”, “2”, “3” etc. “Reference URI” should report the URI – if any – which most closely identifies the image. In the case that the relevant image corresponds to part of a composite image (e.g. figure 2(B) of figure 2), this would be the URI of the composite image e.g. http://toxsci.oxfordjournals.org/content/101/2/239/F2.large.jpg [8]. “Image Name” should report a descriptive name that would allow the image to be uniquely identified

within the corresponding reference (e.g. journal publication). “Permission Obtained for Reuse” should report “Y” (“N”) if permission has (not) been obtained from the copyright holder to publicly redistribute a copy of the corresponding image as part of an ISA-TAB-Nano dataset. “Comment” should record any other miscellaneous information associated with the image.

Table S5: Contents of an “ImageLink” file (“ImageLink_1_for_TOY.article.xls”) created for the “Toy Dataset” discussed in section 6 of the main text and available from the Supporting Information.

Image ID	Reference URI	Image Name	Permission Obtained for Reuse	Comment
1	http://www.made-up-article-address.org/figure3	Figure 3(A)	N	Made up transmission electron microscopy image of nanomaterial sample prepared from TiO2_TOY.article

(19) Any nanomaterial structure representation files, which are not associated with specific Assay file “Measurement Value […]” entries, should be linked to the corresponding Material file using ZIP archives specified in the appropriate “Material Data File” column entry.

All files (e.g. SMILES or CIF files), corresponding to different (partial) representations of a given nanomaterial’s structure, or the structure of a specific component, should be included in a single flat, compressed ZIP archive and the name of this archive should be referred to in the first applicable row of the “Material Data File” column of the relevant Material file. (If the files are intended to represent the structure of a specific component, rather than the nanomaterial as a whole, they should be included in specific ZIP files which should be associated with the “Material Name”

referring to the specific component rather than the initial Material file row(s) referring to the complete nanomaterial [12].) These files should be clearly identified using standard file extensions (e.g. “.smi” for SMILES) and, to reduce ambiguity, the corresponding “Material Data File Description” entry should describe each of the files contained with this ZIP archive.

(20) Empty “Factor Value […]”, “Parameter Value […]” or “Measurement Value […]” columns in Study or Assay files can be deleted without having to update the corresponding Investigation file “Study Protocol Parameters Name”, “Study Factor Name”, or “Study Assay Measurement Name” fields.

Indeed, in general, empty columns can be deleted as long as this does not create orphaned "Unit", "Term Accession Number" or "Term Source REF" columns. Currently, this has to be carried out manually for the NanoPUZZLES Excel based templates.

A disadvantage of allowing for this is that an Investigation file with orphaned “factors”, “parameters” or “measurement names” would give a misleading indication of the (meta)data content of the dataset – although this problem also exists with the generic ISA-TAB-Nano specification if empty columns are not deleted.

(21) Non-applicable columns should be populated with “N/A” where this conveys information.

For example, if the sample described in a physiochemical Study file (e.g. “s_TOY.article_PC.xls” available as part of the “Toy Dataset” in the Supporting Information) was not prepared for an adsorption assay protocol, the “Factor Value [probe]” field, which denotes the “probe” species (e.g. chlorobenzene) for which adsorption to the nanomaterial will be measured [38], should be populated with “N/A”.

In other cases, entering “N/A” would simply be redundant. For example, if a physicochemical measurement was made on a nanomaterial sample for which the Study file “Factor Value [physical state]” entry read “powdered state”, the “Factor Value [medium]” column is clearly non-applicable. However, whilst the existing business rule does not call for redundant “N/A” values to be explicitly entered, in order to reduce the burden of manual curation, possibilities for automated assignment of “N/A” values under these scenarios should be investigated in future work to avoid implying a lack of (meta)data completeness e.g. code might be written which would automatically set a “Factor Value [medium]” entry to “N/A” if the corresponding “Factor Value [physical state]” entry read “powdered state”.

(22) “Measurement Value [statistic(measurement name)]” columns in the templates which use a label of the form “[TO DO:…]” for the statistic or measurement name must either be updated, based on the kind of statistic and/or measurement name indicated by the label(s), or deleted.

For example, “Measurement Value [[TO DO: appropriate average]([TO DO: appropriate size measurement])]” in “a_InvID_PC_size_Method.xls” might be replaced with “Measurement Value [mean of the number distribution(diameter)]” [58]. N.B. In principle, multiple versions of these “generic template columns” might be created within the same Assay file derived from an applicable Assay template file.

Supporting Information Section C. Some Notable Limitations of the NanoPUZZLES Templates and Business Rules Introduced in this Article: In-Depth Discussion

The strengths and weaknesses of the manner in which various challenges associated with the generic ISA-TAB-Nano specification were addressed within NanoPUZZLES were discussed in Supporting Information Section A and possible adaptations of the existing business rules, which should be considered in future work, were discussed in Supporting Information Section B. Likewise, the possibility that the manner in which various kinds of experimental variables are captured via the existing templates may warrant revision in future work was discussed in section 3 of the main text ("Experimental Variables Captured by the Templates"). This section focuses on discussing those issues which are arguably most important to address in future work. Table S 9 summarises these notable limitations. This table is followed by an in-depth explanation of these challenges, along with possible adaptations of the templates and/or business rules which should be considered in future work.

Table S9: Summary of some notable limitations of the NanoPUZZLES templates and business rules.

Limitation no.	Brief description
1	Standardised reporting of stepwise sample preparation is still not handled perfectly.
2	Time dependent physicochemical characterisation data may not be perfectly captured by the templates.
3	Recording of reaction rate constants and quantum yields may need revision.
4	The manner in which chemical composition information is captured via the templates may require revision.
5	There is the possibility of information loss when mapping (raw) data reported in the literature onto predefined “Measurement Value […]” columns.
6	The current templates are not best suited to capturing experimental data for all kinds of samples.
7	The business rules regarding multiple component “characteristics”, “factors” or “parameters” (e.g. mixtures) may require revision.
8	The templates are not currently designed to capture data from *in vivo* toxicology studies.
9	Manually populating the Excel templates is time consuming and error prone.

(1) Standardised reporting of stepwise sample preparation is still not handled perfectly.

The existing NanoPUZZLES approach (see business rule no. 2 in Supporting Information Section B) focused on capturing those experimental variables which were applicable to the most recent sample processing history. However, explicit recording of all stepwise sample preparation steps may be appropriate in future work to better capture experimental variability. Multiple, pre-defined Study file columns (e.g. “Factor

Value [stock suspension Sonication]", "Factor Value [tested suspension Sonication]") might be incorporated within the templates [7]. The creation of multiple "Protocol REF" columns [5] corresponding to "stock suspension preparation", "tested suspension preparation" etc. [7] might also be appropriate. Alternatively, a new business rule might be created stipulating "on-the-fly" creation of multiple versions of pre-defined columns e.g. "Factor Value [Sonication]" could updated to give "Factor Value [Sonication] [treatment order =1]", "Factor Value [Sonication] [treatment order =2]" etc. [5]. The most appropriate means of explicitly recording all stepwise sample preparation steps was under discussion with the ISA-TAB-Nano developers at the time of writing.

(2) Time dependent physicochemical characterisation data may not be perfectly captured by the templates.

Currently, any time dependency of physicochemical measurements is captured via the physicochemical Study file template ("s_InvID_PC.xls") "Factor Value[medium Exposure Duration]" field which calls for the time elapsed since exposure to the medium used to prepare the characterised nanomaterial sample to be recorded. However, in principle, time dependent measurements may be reported which correspond to time points which do not fit this criterion. For example, a "timepoint study" of Murdock et al. [8] involved examining how size and zeta potential values changed over time for samples of copper nanoparticles prepared from refrigerated stock suspensions at different intervals over a one month period. Since sample preparation only entailed dilution, vortexing and warming to room temperature (i.e. the medium was not changed), it was considered legitimate to record the timepoint using the aforementioned "Factor Value[medium Exposure Duration]" field. However, had the medium been changed for experimental testing (as per some of the other measurements reported in Murdock et al. [8]), this would not have been legitimate.

The existing NanoPUZZLES approach would not capture time related metadata concerning the “age” of the nanomaterial sample nor, other than the time spent in the final medium used prior to application of an assay protocol (“Factor Value[medium Exposure Duration]”), the time elapsed between “opening” the received nanomaterial sample and the point in time at which a measurement was made. In part, appropriately capturing these time related metadata would be related to the challenge of appropriately capturing all stepwise sample preparation metadata (limitation no. 1).

Finally, the existing templates and business rules do not address the issue of how to relate the point in time at which physicochemical measurements were made to the point in time at which corresponding biological measurements were made: the *in vitro* cell-based Study file template (“s_InvID_InVitro.CB.xls”) merely contains fields for capturing the time for which the cells were exposed to the nanomaterial sample (“Factor Value[cells Exposure Duration]”) and the time for which the nanomaterial was exposed to the “vehicle” (meaning the final “exposure media” in which the nanomaterial suspension exposed to the cells is prepared) prior to cellular exposure (“Factor Value[vehicle Exposure Duration]”).

(3) Recording of reaction rate constants and quantum yields may need revision.

Currently, reaction rate constants and quantum yields [59] are proposed to be captured via generic “Measurement Value […]” columns (“Measurement Value [mean(rate constant)]”, “Measurement Value [mean(quantum yield)]”) in the relevant Assay file template (“a_InvID_PC_reactivity.rateofchange_of.X_SeparationTechnique_Method.xls”) with their identities captured via corresponding “Parameter Value […]” and “Comment […]” columns which, in the latter case, will only contain free text entries. This is

illustrated in Table S 10 for the populated version of this template created for the “Toy Dataset” described in section 6 of the main text: only the capturing of quantum yield data using this template, based on the study of photochemical production of hydrogen peroxide by Hoffman et al. [59], is illustrated, but the manner in which rate constant data would be captured would be analogous i.e. the “Comment [type of rate constant]” and “Measurement Value [mean(rate constant)]” columns would be used instead of the “Comment [type of quantum yield]” and “Measurement Value [mean(quantum yield)]” columns.

Table S10: Recording of quantum yield information in the “Toy Dataset” (described in section 6 of the main text) reactivity Assay file (“a_TOY.article_PC_reactivity.rateofchange_of.H2O2_CapillaryColumnSeparation.xls”) presented in the Supporting Information (c.f. Hoffman et al. [59]). N.B. Only the columns relevant to the current discussion are shown, not including the “Term Accession Number” and “Term Source REF” columns used to link “Parameter Value […]” entries to terms from ontologies, and sample names have been abbreviated due to space constraints.

Sample Name	Comment [type of quantum yield]	Protocol REF	Parameter Value [analyte role]	Measurement Value [mean (quantum yield)]	Unit
s_..._ derived: 7,8,9	peroxide production	reactivity based on analysis of hydrogen peroxide separated by capillary column separation, measured by gas chromatography-mass spectrometry	hydrogen peroxide	3.4	percent

However, whilst this allows for flexibility, revision of this approach may be appropriate to promote standardised recording and facilitate automated computational analysis. The use of the “Comment [type of quantum yield]” or “Comment [type of rate constant]” columns to identify the nature of the “quantum yield” or “rate constant” recorded in the relevant “Measurement Value […]” column is problematic for two reasons: (1) “Comment […]” fields can only be populated with free text entries, rather than allowing links to ontologies to be created [5,27]; (2) since these “Comment […]” fields would be associated with the corresponding “Sample Name” field [5], this would not allow more than one kind of quantum yield (e.g. quantum yield values for peroxide production and peroxide destruction [59]), or rate constant, to be associated with a given “Sample Name” identifier and still be differentiated.

One possible means of addressing this in future work would be to add “Measurement Value [*measurement name*]” columns with very specific “measurement name” titles e.g. “Measurement Value [quantum yield for peroxide destruction]” and “Measurement Value [quantum yield for peroxide creation]”. In principle, although possibly not in practice, this would allow these names to be linked to terms from ontologies via the Investigation file “Study Assay Measurement Name” field [3]. One further point which is worth noting here is that future adaptations of the reactivity Assay file might also employ the adaptation of the ToxBank approach [19,56] for linking to “derived data points” (e.g. dose-response curve statistics or, in the current context, quantum yield estimates), instead of the current NanoPUZZLES business rule (rule no. 10) based on “derived sample” identifiers, which was discussed when explaining NanoPUZZLES business rule no. 10 in Supporting Information Section B. However, the exact manner in which the NanoPUZZLES approach to capturing reactivity data should be revised in future work requires further consideration and discussion with the ISA-TAB-Nano developers.

(4) The manner in which chemical composition information is captured via the templates may require revision.

As discussed in Supporting Information Section A (challenge no. 2), the most appropriate means of recording certain kinds of nanomaterial chemical composition information using ISA-TAB-Nano is arguably not clear. In particular, the templates (see section 3 of the main text) and business rules 5- 8 (Supporting Information Section B) developed within NanoPUZZLES sought to address how best to record the following kinds of information: (a) experimentally determined (or verified) intrinsic chemical composition information and (b) specific kinds of composition information such as (1) the suspension medium in which the tested nanomaterials were originally received (if any) or (2) “impurities”.

The handling of experimentally determined intrinsic chemical composition information may require revision as the approach developed within NanoPUZZLES stipulates that all such information should be recorded within the Material file, even in the case of specific “Characteristics [...]” columns introduced to capture certain kinds of chemical composition information ("Characteristics [component proportion]","Characteristics [Product impurities found {MEDDRA: http://purl.bioontology.org/ontology/MDR/10069178}]","Characteristics [Impurities proportions]","Characteristics[purity {NPO: http://purl.bioontology.org/ontology/npo#NPO_1345}]"), in contrast to the standard ISA-TAB-Nano approach (as of version 1.2) to experimentally determined characteristics which stipulates these should be recorded via Assay files [11,12]. Whilst the approach developed within NanoPUZZLES resolves potential ambiguity regarding where experimentally determined intrinsic chemical composition data recorded using specific Material field types other than “Characteristics […]” columns, such as the “Material Chemical Name” field [12], should be recorded and ensures

that all intrinsic chemical composition information is recorded in the same place (i.e. the same Material file), this only allows experimentally determined or verified intrinsic composition information to be distinguished via free text entries in “Comment [...]” fields, which would currently need to be added “on-the-fly” at the point of data curation, and via documenting this information using the “Material Description” field. Hence, it might be appropriate to record experimentally determined intrinsic composition information using new Assay file templates *in addition* to summarising all composition information in the Material file. This would enable the corresponding experimental conditions (e.g. medium) and technique (e.g. X-ray photoelectron spectroscopy) [8] to be documented in a standardised fashion for all experimentally determined composition information using the applicable Assay file, Study file and Investigation file fields as per all other experimentally determined physiochemical properties discussed in section 3 of the main text. For example, an experimentally identified dispersant aid [8] might be captured via a new Assay file “Measurement Value [chemical component identified]” column as well as being documented, using the “Material Chemical Name” and “Material Type” fields of the Material file [12]. Whether or not experimentally determined values for the specific “Characteristics […]” fields introduced in NanoPUZZLES to capture certain kinds of chemical composition information ("Characteristics [component proportion]","Characteristics [Product impurities found {MEDDRA: http://purl.bioontology.org/ontology/MDR/10069178}]","Characteristics [Impurities proportions]","Characteristics[purity {NPO: http://purl.bioontology.org/ontology/npo#NPO_1345}]") should also be recorded using novel Assay file templates *and* be summarised in these Material file fields, in contrast to the standard ISA-TAB-Nano approach (as of version 1.2) that Material file

“Characteristics [….]” fields should only refer to nominal (or vendor supplied) values, is an open question.

One point which requires further consideration here is how the link between characteristics which are applicable to specific nanomaterial components (e.g. “Characteristics [component proportion]”), rather than the nanomaterial as a whole, would best be documented if experimentally determined/confirmed values for these characteristics were (only) recorded via Assay files. Indeed, this issue is also relevant for recording of experimentally determined/confirmed values for certain kinds of intrinsic chemical composition information which would otherwise be recorded using other kinds of Material file fields such as “Material Chemical Name” values for specific components. Hence, revision of the NanoPUZZLES approach for capturing experimentally determined/confirmed intrinsic chemical composition information would require further discussions with the ISA-TAB-Nano developers.

Since the classification of certain constituents as “impurities” might be somewhat subjective, it would arguably be more appropriate in future work to treat all impurities as per any other chemical component i.e. record them as separate rows in the Material file with the “Material Type” field annotations for that row including the label “impurity”. This would also entail removing the "Characteristics [Product impurities found {MEDDRA: http://purl.bioontology.org/ontology/MDR/10069178}]" and “Characteristics [Impurities Proportions]” fields. Whilst this would make the Material files less compact than the approach developed within NanoPUZZLES (since more rows would need to be added to the files), it would avoid the structure of the files changing depending upon whether or not certain researchers considered a given component to be an “impurity” as well as enabling information regarding the nature of linkages (e.g. covalent) between “impurities” and major components to be captured via the Material file “Material Linkage Type” field [12].

Finally, the existing NanoPUZZLES templates and business rules are perhaps not best suited for capturing all kinds of percentage composition information. For instance, consider a nanomaterial core which was 90% Fe_2O_3 and 10% TiO_2 [60]. According to the current NanoPUZZLES approach, this kind of information would be addressed by treating the minor component as an "impurity" of the core (to be recorded via the "Characteristics [Product impurities found {MEDDRA: http://purl.bioontology.org/ontology/MDR/10069178}]" and "Characteristics [Impurities proportions]" column entries associated with the row describing the nanomaterial core), if it was described as an "impurity" in the original publication from which the data were extracted. If the minor component was not described as an "impurity", both constituents would be treated as separate components and described using separate rows of the Material file, in keeping with the standard ISA-TAB-Nano approach [12], each with the same "Material Type" annotation: "core constituent". In the latter case, the best manner in which to capture the percentage composition information, if the nanomaterial comprised more than one type of a component (e.g. core and shell), remains unclear. One possibility might be to populate the corresponding "Characteristics [component proportion]" entries with the percentage values for the specific component type and use very specific unit terms in the corresponding "Unit" column entries e.g. "percentage contribution to the core".

(5) There is the possibility of information loss when mapping (raw) data reported in the literature onto predefined "Measurement Value […]" columns.

The use of predefined "Measurement Value […]" columns which closely correspond to the values which modellers might wish to predict (e.g. "Measurement Value [mean(percent cytotoxicity)]" [61,62]) is arguably of value for end users of the data collection i.e. it reduces the amount of interpretation and/or processing of the data

required by modellers. However, since the data reported in the literature may not directly correspond to these predefined columns, some interpretation and/or processing of the data might be required during data collection. Both incorrect interpretations or calculations may lead to errors in the curated data. The possibility of information loss due to incorrect interpretation is illustrated via the following example. Since the “z-average size” (“size” meaning “hydrodynamic diameter”) is considered the “primary and most stable parameter” obtained from dynamic light scattering [63], it might be considered reasonable to record reported “size” (or “average size”) values from dynamic light scattering using the “Measurement Value [z-average(hydrodynamic diameter)]” column, in the NanoPUZZLES “a_InvID_size_DLS.xls” Assay file template, if no further details are provided in the publication from which the data were extracted [64]. However, this interpretation of the data may be erroneous: other kinds of average (such as the number weighted average) hydrodynamic diameter may be obtained from dynamic light scattering and, indeed, may be more appropriate if the nanomaterial sample does not have a unimodal size distribution i.e. there is more than one peak [58,63].

The possibility of information loss via incorrect calculations is illustrated via the following example. If mean percent viability values (normalised to control), are provided [8], populating the pre-defined “Measurement Value [mean(percent cytotoxicity)]” column, in the NanoPUZZLES “a_InvID_cytotoxicity.cell-viability_Method.xls” Assay file template, entails subtracting these values from 100 [62], which might be carried out incorrectly.

Within the context of the NanoPUZZLES project, the use of “Comment […]” columns was advocated to describe any necessary interpretation and/or processing that took place when populating the relevant Assay file templates. However, it is arguable that stipulating the mandatory recording of all originally reported values used to derive

predefined “Measurement Value […]” column entries (e.g. via additional “Measurement Value […]” fields created “on-the-fly”), along with saving the calculation steps in the Excel version of the datasets, would further reduce potential loss of information due to calculation errors and make any calculation errors and possible data misinterpretations clear to the end user of the dataset. In keeping with this, it may be appropriate to adopt the approach employed by the ToxBank ISA-TAB Study file template [18–20] for denoting positive/negative control samples which might, in the current context, be associated with new “Measurement Value [….]” fields corresponding to positive/negative control data used to calculate the values entered in the pre-defined “Measurement Value […]” columns.

Nonetheless, it should be noted that, in some cases, mapping of data reported in publications onto the predefined “Measurement Value […]” columns defined in the NanoPUZZLES Assay templates will simply not be possible. For example, it may be necessary to create new “Measurement Value […]” columns for number weighted average hydrodynamic diameters in Assay files prepared using the NanoPUZZLES “a_InvID_size_DLS.xls” template – either “on-the-fly”, during data curation, or via adding new predefined columns to this template [58].

(6) The current templates are not best suited to capturing experimental data for all kinds of samples.

The NanoPUZZLES Assay file templates referred to in this article are designed to record measured data (either raw or derived) which are associated with samples corresponding to a tested nanomaterial denoted via the Study file “Source Name” or “Factor Value [nanomaterial]” entry, for a physicochemical or biological study respectively. However, this is not best suited for recording data for chemicals of interest without dimensions in the nanoscale e.g. if comparing the effects of microsized to nanosized particles [54] or if testing a small molecule positive control

[55]: a more general name, other than “Factor Value [nanomaterial]”, would be more appropriate. This would be particularly appropriate for recording (raw) data associated with positive/negative control samples, denoted using the ToxBank ISA-TAB Study file template approach [18–20], as proposed above.

(7) The business rules regarding multiple component “characteristics”, “factors” or “parameters” (e.g. mixtures) may require revision.

The application of NanoPUZZLES business rules 3 and 4 (Supporting Information Section B), which stipulate that multiple component entries in these field types should be populated using semi-colon delimited lists and that the entries in corresponding fields should be populated using corresponding semi-colon delimited lists, would lead to some column entries being populated with mutually exclusive values. These entries would not have a clear semantic interpretation if they were parsed in isolation. For example, the *in vitro* cell-based Study file in the “Toy Dataset” discussed in section 6 of the main text and available from the Supporting Information (“s_TOY.article_InVitro.CB.xls”) contains a “Factor Value [vehicle serum]” entry “fetal bovine serum; horse serum”: the corresponding “Factor Value [vehicle serum heat treatment]” entry was “TRUE;FALSE”. The entry “TRUE;FALSE” is comprised of mutually exclusive values and this entry has no clear semantic meaning if parsed in isolation.

This could pose a problem when trying to parse the datasets generated according to these business rules. Indeed, the current versions of the ISA-Tools [21,22], which might be extended to parse ISA-TAB-Nano files in the future, would not be able to interpret any kinds of field entries where multiple component “characteristics”, “factors”, or “parameters” were treated as semi-colon delimited lists: these would be treated as simple strings [28]. This is also true for the current implementation of the

nanoDMS database system discussed at the end of section 7 of the main text [17,65,66].

Ensuring that software designed to parse ISA-TAB-Nano files could take account of these business rules would be necessary to take full advantage of the conversion to linked data [67]. However, since these business rules have been clearly documented, this does provide the basis for determining a possible solution: the development of a parser which was able to recognise (1) that semi-colon delimited “characteristics”, “factors”, or “parameters” refer to multiple components and that (2) that multiple component entries in (explicitly specified) corresponding fields should not be parsed in isolation.

An additional element of complexity which would need to be taken into account if implementing these business rules in parsing software concerns some corresponding fields which *can* be assigned multiple component entries that have *partial* semantic meaning if parsed in isolation. For instance, consider a “Factor Value [vehicle]” entry populated with a complex mixture and the corresponding entry for “Factor Value [vehicle volume]”. For example, some of the samples prepared prior to assessment via the Ames test in the “Toy Dataset” file “s_TOY.article_InVitro.CB.xls” contained “Factor Value [vehicle]” entries of “Oxoid nutrient broth; deionized water; S9 mix; molten top agar”, reflecting the complex mixtures which might constitute the final exposure medium in which the nanomaterial suspension exposed to the cells is prepared [55]. For this scenario, the volume proportion of the different liquid constituents might be valuable information [55]. This information can be captured via populating the corresponding field “Factor Value [vehicle volume]” with the corresponding volumes, in keeping with the existing NanoPUZZLES business rule no. 4 (Supporting Information Section B), i.e. by populating this field with

“0.1;0.5;0.5;2” and the corresponding “Unit” column with “milliliter; milliliter; milliliter; milliliter”: this was carried out for the “Toy Dataset”.

However, since the sum of these values would also, in principle, be a semantically meaningful entry, this could also be entered in “Factor Value [vehicle volume]” if the volume proportions of the different mixture components specified in “Factor Value [vehicle]” were not provided but, instead, merely the overall volume of the mixture was available. Under this scenario, the cumulative volume and its corresponding unit (i.e. “3.1” and “milliliter" for the example considered here) would entered in the “Factor Value [vehicle volume]” field and the corresponding “Unit” column respectively.

Hence, any software parsing these fields would need to recognise that, for explicitly specified fields such as “Factor Value [vehicle]”, an entry of “0.1;0.5;0.5;2” was equivalent to “3.1”.

(8) The templates are not currently designed to capture data from *in vivo* toxicology studies.

Various published nanotoxicology studies have presented *in vivo* toxicity data in recent years [54,68,69]. At the time of writing, a Study file template, for capturing sample preparation variables, and Assay file templates, for capturing data associated with key endpoints such as mortality, for *in vivo* assays were under development within the NanoPUZZLES project.

(9) Manually populating the Excel templates is time consuming and error prone.

Whilst using Excel-based templates offers the advantage of allowing nanotoxicologists involved in data curation to continue to work with software they are most likely familiar with [70], the need to manually populate the templates may outweigh this advantage. Manually populating the templates means that

corresponding field entries in different files need to be duplicated, or that information needs to be essentially duplicated, by hand. For example, all “Sample Name” entries in a Study file need to be manually copied across to the appropriate Assay file “Sample Name” column. As another example, all Assay “measurement name” values and “statistic” values (unless they were amongst those statistic names predefined in the Investigation file template) need to be copied across to the corresponding Investigation file “Study Assay Measurement Name” and “Comment [Statistic name]” fields. In addition, Investigation file fields which reference corresponding Study, Assay or Material file names must also be manually populated.

Likewise, any redundant “N/A” values (e.g. a “Factor Value [medium]” entry when the corresponding “Factor Value [physical state]” field value is “powdered state”) would need to be manually entered, hence the existing NanoPUZZLES business rules (see Supporting Information Section B, rule no. 21) allow for such entries to be skipped to reduce the burden of manual curation. However, this could misleadingly imply a lack of (meta)data completeness. The possibility of auto-generating these “N/A” values should certainly be explored in future work e.g. code might be written which would automatically set a “Factor Value [medium]” entry to “N/A” if the corresponding “Factor Value [physical state]” entry read “powdered state”.

As a related issue, if empty columns were deleted from Study, Assay or Material files, any dependent columns (e.g. “Unit”, “Term Accession Number”, “Term Source REF”) would currently need to be manually deleted within the NanoPUZZLES templates.

Finally, manually populating these files means that fields linking to ontologies need to be manually populated. For example, “preferred name”, “Term Accession Number” and “Term Source REF” values need to be manually copied and pasted from, say, BioPortal [24,25] unless these values are amongst those which have been prepopulated either as hardcoded values or as corresponding drop down lists e.g. as

per the Investigation file template “Study Protocol Parameters Name” with its corresponding “Study Protocol Parameters Name Term Accession Number” and “Study Protocol Parameters Name Term Source REF” fields.

In addition to being time consuming, carrying out the described steps manually also increases the chance of transcription errors. In the case of the manually predefined ontologies in the Investigation file template “ONTOLOGY SOURCE REFERENCE” section, there is also a risk that the hardcoded “Term Source Version” entries may not correspond to the version of the ontology from which terms are manually retrieved via BioPortal during data collection.

One potential possibility for addressing the challenges related to making use of ontologies *might* be to extend the NanoPUZZLES Excel templates using the “RightField” software [70,71]. However, this possibility remains to be investigated. Alternatively, if the ISAcreator software program [21,22] was extended, the NanoPUZZLES Excel templates might serve as the basis for XML templates to be used for creating ISA-TAB-Nano datasets using this program. N.B. This possibility was not an option at the time of writing, since the ISAcreator software program had not been extended to allow for the creation of ISA-TAB-Nano files.

References

1. Thomas, D. G.; Gaheen, S.; Harper, S. L.; Fritts, M.; Klaessig, F.; Hahn-Dantona, E.; Paik, D.; Pan, S.; Stafford, G. A.; Freund, E. T.; Klemm, J. D.; Baker, N. A. *BMC Biotechnol.* **2013**, *13*, 2.
2. ISA-TAB-Nano Wiki: Assay File Documentation https://wiki.nci.nih.gov/display/ICR/Assay (accessed Mar 28, 2015).
3. ISA-TAB-Nano Wiki: Investigation File Documentation https://wiki.nci.nih.gov/display/ICR/Investigation (accessed Mar 28, 2015).
4. Rayner, T. F.; Rocca-Serra, P.; Spellman, P. T.; Causton, H. C.; Farne, A.; Holloway, E.; Irizarry, R. A.; Liu, J.; Maier, D. S.; Miller, M.; Petersen, K.; Quackenbush, J.; Sherlock, G.; Stoeckert, C. J.; White, J.; Whetzel, P. L.; Wymore, F.; Parkinson, H.; Sarkans, U.; Ball, C. A.; Brazma, A. *BMC Bioinformatics* **2006**, *7*, 489.
5. Rocca-Serra, P.; Sansone, S.-A.; Brandizi, M.; Hancock, D.; Harris, S.; Lister, A.; Miller, M.; O'Neill, K.; Taylor, C.; Tong, W. Specification documentation: release candidate 1, ISA-TAB 1.0 http://isatab.sourceforge.net/docs/ISA-TAB_release-candidate-1_v1.0_24nov08.pdf (accessed Jul 21, 2015).
6. STATistics Ontology (STATO) Homepage http://stato-ontology.org/ (accessed Aug 3, 2015).
7. *Guidance on Sample Preparation and Dosimetry for the Safety Testing of Manufactured Nanomaterials*; Series on the Safety of Manufactured Nanomaterials; 36; Organisation for Economic Co-operation and Development, 2012.
8. Murdock, R. C.; Braydich-Stolle, L.; Schrand, A. M.; Schlager, J. J.; Hussain, S. M. *Toxicol. Sci.* **2008**, *101*, 239–253.
9. Ostraat, M. L.; Mills, K.; Guzan, K.; Murry, D. *Int. J. Nanomedicine* **2013**, 7.
10. Lynch, I.; Dawson, K. A. *Nano Today* **2008**, *3*, 40–47.
11. ISA-TAB-Nano 1.2 Release Notes https://wiki.nci.nih.gov/display/ICR/ISA-TAB-Nano+1.2+Release+Notes (accessed Mar 28, 2015).
12. ISA-TAB-Nano Wiki: Material File Documentation https://wiki.nci.nih.gov/display/ICR/Material (accessed Mar 28, 2015).
13. Zhu, H.; Hu, H.; Wang, Z.; Zuo, D. *Nanoscale Res. Lett.* **2009**, *4*, 1009.
14. Thomas A. J. Kuhlbusch, H. F. K. *NanoCare : Health related aspects of nanomaterials (Final Scientific Report)*; Final Scientific Report; NanoCare Project, 2009; p. 151.
15. ISA-TAB-Nano Wiki: Study File Documentation https://wiki.nci.nih.gov/display/ICR/Study (accessed Mar 28, 2015).
16. MODERN Project Tools http://modern-fp7.biocenit.cat/tools.html (accessed Mar 28, 2015).
17. Rallo, R. An ISA-TAB Nano compliant data management system for nanosafety modelling https://nciphub.org/resources/500 (accessed Mar 27, 2015).
18. ToxBank Project Homepage http://www.toxbank.net/ (accessed Mar 28, 2015).
19. Kohonen, P.; Benfenati, E.; Bower, D.; Ceder, R.; Crump, M.; Cross, K.; Grafström, R. C.; Healy, L.; Helma, C.; Jeliazkova, N.; Jeliazkov, V.; Maggioni, S.; Miller, S.; Myatt, G.; Rautenberg, M.; Stacey, G.; Willighagen, E.; Wiseman, J.; Hardy, B. *Mol. Inform.* **2013**, *32*, 47–63.
20. ToxBank ISA-TAB Templates https://github.com/ToxBank/isa2rdf/tree/master/isa2rdf/isa2rdf-cli/src/main/resources/toxbank-config (accessed Mar 28, 2015).
21. ISA-Tools Software http://www.isa-tools.org/software-suite/ (accessed Jul 21, 2015).

22. Rocca-Serra, P.; Brandizi, M.; Maguire, E.; Sklyar, N.; Taylor, C.; Begley, K.; Field, D.; Harris, S.; Hide, W.; Hofmann, O.; Neumann, S.; Sterk, P.; Tong, W.; Sansone, S.-A. *Bioinformatics* **2010**, *26*, 2354–2356.
23. Thomas, D. G.; Pappu, R. V.; Baker, N. A. *J. Biomed. Inform.* **2011**, *44*, 59–74.
24. Whetzel, P. L.; Noy, N. F.; Shah, N. H.; Alexander, P. R.; Nyulas, C.; Tudorache, T.; Musen, M. A. *Nucleic Acids Res.* **2011**, *39*, W541–W545.
25. BioPortal Homepage http://bioportal.bioontology.org/ (accessed Mar 28, 2015).
26. ISA-TAB-Nano Wiki: Curated Examples https://wiki.nci.nih.gov/display/ICR/ISA-TAB-Nano+Curated+Examples (accessed Jul 21, 2015).
27. ISA-TAB-Nano Wiki: Assay File Examples https://wiki.nci.nih.gov/display/ICR/Assay+File+Examples (accessed Aug 3, 2015).
28. Rocca-Serra, P. Private Communication.
29. ISAforum: How to encode multiple values https://groups.google.com/forum/#!searchin/isaforum/mixture/isaforum/HOTScd3EeDY/1EIjmtOhnUsJ (accessed Mar 28, 2015).
30. Weininger, D. *J. Chem. Inf. Comput. Sci.* **1988**, *28*, 31–36.
31. Daylight Theory Manual (Version 4.9): 3. SMILES - A Simplified Chemical Language http://www.daylight.com/dayhtml/doc/theory/theory.smiles.html (accessed Mar 28, 2015).
32. Toropova, A. P.; Toropov, A. A.; Benfenati, E.; Gini, G.; Leszczynska, D.; Leszczynski, J. *Mol. Divers.* **2011**, *15*, 249–256.
33. Gentleman, D. J.; Chan, W. C. W. *Small* **2009**, *5*, 426–431.
34. Toropova, A. P.; Toropov, A. A. *Chemosphere* **2013**, *93*, 2650–2655.
35. Hall, S. R.; Allen, F. H.; Brown, I. D. *Acta Crystallogr. A* **1991**, *47*, 655–685.
36. Puzyn, T.; Rasulev, B.; Gajewicz, A.; Hu, X.; Dasari, T. P.; Michalkova, A.; Hwang, H.-M.; Toropov, A.; Leszczynska, D.; Leszczynski, J. *Nat. Nanotechnol.* **2011**, *6*, 175–178.
37. Thomas, D. G.; Chikkagoudar, S.; Chappell, A. R.; Baker, N. A. In *2012 IEEE International Conference on Bioinformatics and Biomedicine Workshops (BIBMW)*; 2012; pp. 889–894.
38. Chen, R.; Zhang, Y.; Darabi Sahneh, F.; Scoglio, C. M.; Wohlleben, W.; Haase, A.; Monteiro-Riviere, N. A.; Riviere, J. E. *ACS Nano* **2014**, *8*, 9446–9456.
39. ISA-TAB-Nano Wiki https://wiki.nci.nih.gov/display/ICR/ISA-TAB-Nano (accessed Mar 27, 2015).
40. Hu, X.; Cook, S.; Wang, P.; Hwang, H. *Sci. Total Environ.* **2009**, *407*, 3070–3072.
41. Stefaniak, A. B.; Hackley, V. A.; Roebben, G.; Ehara, K.; Hankin, S.; Postek, M. T.; Lynch, I.; Fu, W.-E.; Linsinger, T. P. J.; Thünemann, A. F. *Nanotoxicology* **2013**, *7*, 1325–1337.
42. Lynch, I.; Weiss, C.; Valsami-Jones, E. *Nano Today* **2014**, *9*, 266–270.
43. Experimental Factor Ontology: Cell Line Definition http://www.ebi.ac.uk/efo/EFO_0000322 (accessed Mar 28, 2015).
44. Dearden, J. C.; Cronin, M. T. D.; Kaiser, K. L. E. *SAR QSAR Environ. Res.* **2009**, *20*, 241–266.
45. Lubinski, L.; Urbaszek, P.; Gajewicz, A.; Cronin, M. T. D.; Enoch, S. J.; Madden, J. C.; Leszczynska, D.; Leszczynski, J.; Puzyn, T. *SAR QSAR Environ. Res.* **2013**, *24*, 995–1008.
46. *Guidance Manual for the Testing of Manufactured Nanomaterials: OECD's Sponsorship Programme; First Revision*; Series of Safety of Manufactured Nanomaterials; 25; Organisation for Economic Co-operation and Development, 2010.
47. OECD Harmonised Templates for Reporting Chemical Test Summaries http://www.oecd.org/ehs/templates/templates.htm (accessed Mar 28, 2015).

48. OECD. *OECD Principles on Good Laboratory Practice*; Organisation for Economic Co-operation and Development: Paris, 1998.
49. Aberg, C. NanoSafety Cluster Databases Working Group. Overview and recommendation of data quality: Working draft http://www.nanosafetycluster.eu/working-groups/4-database-wg/tasks-2/2013-2.html (accessed Mar 20, 2015).
50. Lewis, R. W.; Billington, R.; Debryune, E.; Gamer, A.; Lang, B.; Carpanini, F. *Toxicol. Pathol.* **2002**, *30*, 66–74.
51. BioAssay Ontology LC50 Definition http://bioportal.bioontology.org/ontologies/BAO?p=classes&conceptid=http%3A%2F%2Fwww.bioassayontology.org%2Fbao%23BAO_0002145 (accessed Mar 20, 2015).
52. OECD. *Test No. 471: Bacterial Reverse Mutation Test*; Organisation for Economic Co-operation and Development: Paris, 1997.
53. Doak, S. H.; Manshian, B.; Jenkins, G. J. S.; Singh, N. *Mutat. Res. Toxicol. Environ. Mutagen.* **2012**, *745*, 104–111.
54. Golbamaki, N.; Rasulev, B.; Cassano, A.; Robinson, R. L. M.; Benfenati, E.; Leszczynski, J.; Cronin, M. T. D. *Nanoscale* **2015**, *7*, 2154–2198.
55. Shinohara, N.; Matsumoto, K.; Endoh, S.; Maru, J.; Nakanishi, J. *Toxicology Letters* **2009**, *191*, 289–296.
56. 4.3. Step-by-step instructions for creating datasets (dose-response) | ToxBank http://www.toxbank.net/tutorials/isa-tab/4-step-step-dose-response3 (accessed Jul 3, 2015).
57. NanoParticle Ontology (NPO): titanium oxide nanoparticle http://bioportal.bioontology.org/ontologies/NPO?p=classes&conceptid=http%3A%2F%2Fpurl.bioontology.org%2Fontology%2Fnpo%23NPO_1486 (accessed Jul 30, 2015).
58. Baalousha, M.; Lead, J. R. *Environ. Sci. Technol.* **2012**, *46*, 6134–6142.
59. Hoffman, A. J.; Carraway, E. R.; Hoffmann, M. R. *Environ. Sci. Technol.* **1994**, *28*, 776–785.
60. Walser, T.; Studer, C. *Regul. Toxicol. Pharmacol.* **2015**, *72*, 569–571.
61. Toropova, A. P.; Toropov, A. A.; Benfenati, E.; Korenstein, R. *J. Nanoparticle Res.* **2014**, *16*, 2282.
62. BioAssay Ontology Percent Cytotoxicity Definition http://bioportal.bioontology.org/ontologies/BAO?p=classes&conceptid=http%3A%2F%2Fwww.bioassayontology.org%2Fbao%23BAO_0000006 (accessed Mar 20, 2015).
63. *Dynamic light scattering - common terms defined (Version 1 Whitepaper)*; Malvern Instruments Ltd., 2014.
64. Sayes, C.; Ivanov, I. *Risk Anal.* **2010**, *30*, 1723–1734.
65. Pons, R.; Cester, J.; Giralt, F.; Rallo, R. *D2.1. MODERN Data Repository*; European Union Seventh Framework Programme Project Deliverable Report D2.1; 2014.
66. Nanomaterial Data Management System (nanoDMS) http://biocenitc-deq.urv.cat/nanodms (accessed Mar 29, 2015).
67. González-Beltrán, A.; Maguire, E.; Sansone, S.-A.; Rocca-Serra, P. *BMC Bioinformatics* **2014**, *15*, S4.
68. Harper, S. L.; Carriere, J. L.; Miller, J. M.; Hutchison, J. E.; Maddux, B. L. S.; Tanguay, R. L. *ACS Nano* **2011**, *5*, 4688–4697.
69. Landsiedel, R.; Ma-Hock, L.; Hofmann, T.; Wiemann, M.; Strauss, V.; Treumann, S.; Wohlleben, W.; Gröters, S.; Wiench, K.; van Ravenzwaay, B. *Part. Fibre Toxicol.* **2014**, *11*, 16.
70. Wolstencroft, K.; Owen, S.; Horridge, M.; Krebs, O.; Mueller, W.; Snoep, J. L.; Preez, F. du; Goble, C. *Bioinformatics* **2011**, *27*, 2021–2022.
71. RightField Software Homepage http://www.rightfield.org.uk/ (accessed Mar 29, 2015).
